# Identification of Putative Genes Involved in Limonoids Biosynthesis in Citrus by Comparative Transcriptomic Analysis

**DOI:** 10.3389/fpls.2017.00782

**Published:** 2017-05-12

**Authors:** Fusheng Wang, Mei Wang, Xiaona Liu, Yuanyuan Xu, Shiping Zhu, Wanxia Shen, Xiaochun Zhao

**Affiliations:** ^1^Citrus Research Institute, Southwest University and Chinese Academy of Agricultural SciencesChongqing, China; ^2^National Citrus Engineering Research CenterChongqing, China; ^3^College of Horticulture and Landscape Architecture, Southwest UniversityChongqing, China; ^4^Chongqing Yongchuan Institute for Food and Drug ControlChongqing, China

**Keywords:** biosynthesis of limonoids, transcriptome, digital gene expression profiling, VIGS, *CiOSC*

## Abstract

Limonoids produced by citrus are a group of highly bioactive secondary metabolites which provide health benefits for humans. Currently there is a lack of information derived from research on the genetic mechanisms controlling the biosynthesis of limonoids, which has limited the improvement of citrus for high production of limonoids. In this study, the transcriptome sequences of leaves, phloems and seeds of pummelo (*Citrus grandis* (L.) Osbeck) at different development stages with variances in limonoids contents were used for digital gene expression profiling analysis in order to identify the genes corresponding to the biosynthesis of limonoids. Pair-wise comparison of transcriptional profiles between different tissues identified 924 differentially expressed genes commonly shared between them. Expression pattern analysis suggested that 382 genes from three conjunctive groups of K-means clustering could be possibly related to the biosynthesis of limonoids. Correlation analysis with the samples from different genotypes, and different developing tissues of the citrus revealed that the expression of 15 candidate genes were highly correlated with the contents of limonoids. Among them, the cytochrome P450s (*CYP450s*) and transcriptional factor *MYB* demonstrated significantly high correlation coefficients, which indicated the importance of those genes on the biosynthesis of limonoids. *CiOSC* gene encoding the critical enzyme oxidosqualene cyclase (OSC) for biosynthesis of the precursor of triterpene scaffolds was found positively corresponding to the accumulation of limonoids during the development of seeds. Suppressing the expression of *CiOSC* with VIGS (Virus-induced gene silencing) demonstrated that the level of gene silencing was significantly correlated to the reduction of limonoids contents. The results indicated that the *CiOSC* gene plays a pivotal role in biosynthesis of limonoids.

## Introduction

Citrus is the largest fruit crop in the world, with over 4,000 years history of cultivation. Limonoids are one of the most important classes of phytochemicals predominantly produced in the Rutaceae, Meliaceae, Cneoraceae, and Simaroubaceae families, with citrus being the only one among them that produce edible fruits. Evaluation of the biological activity of citrus limonoids has revealed the potential of these compounds in anticancer, antimicrobial, antioxidant and insecticidal capacities (Alford and Murray, 2000; Chowdhury et al., 2003; Ono et al., 2011; Kim et al., 2013). Limonoids exist in a number of plant organs and tissues such as stems, leaves, seeds and fruit tissues (McIntosh et al., 1982; Rouseff and Nagy, 1982; Herman et al., 1989; Li et al., 2014). The accumulation of limonoids is influenced by genotype, environment, the type of tissues and developmental stages of the plant (Rouseff and Nagy, 1982; Baldwin et al., 2010; Chebrolu et al., 2012; Li et al., 2014; Chen et al., 2015; Wang et al., 2016).

**FIGURE 1:**
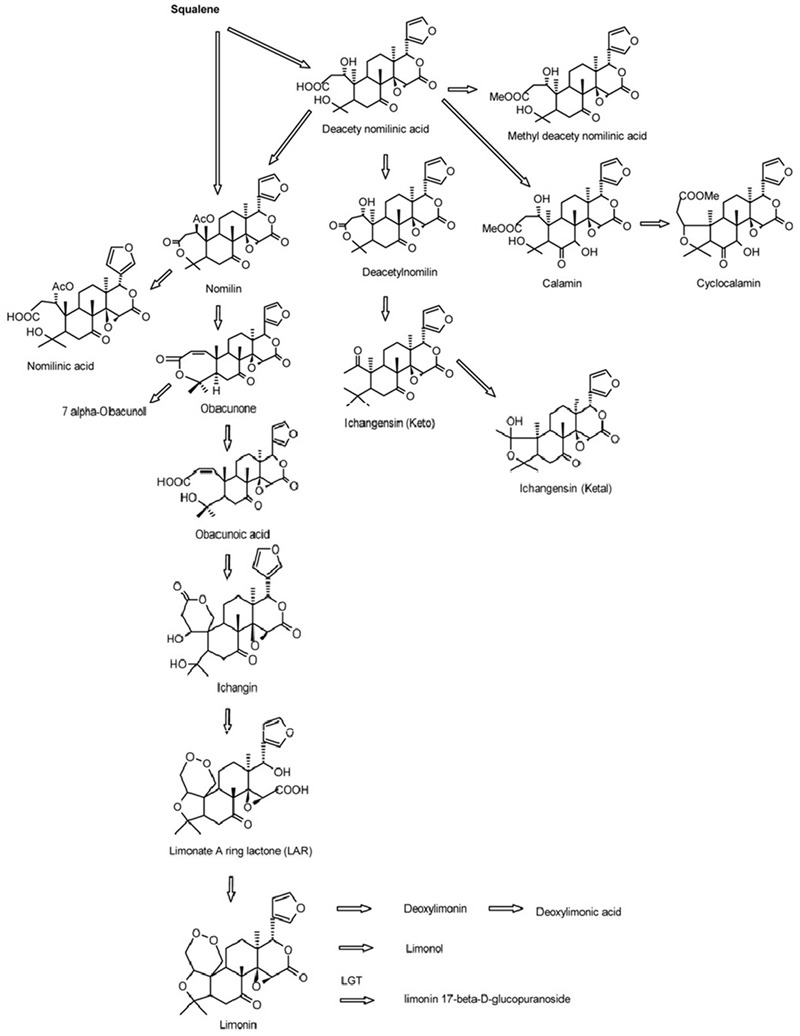
**Biosynthetic pathways of limonoids in citrus (Roy and Saraf, 2006)**.

Limonoids are synthesized via the isoprenoids biosynthetic pathway, starting with cyclization of squalene (Roy and Saraf, 2006). In triterpenoids biosynthetic pathway, oxidosqualene cyclases (OSCs) are the first committed enzymes, which convert triterpenoid carbon structures into precursors of triterpenoid metabolites (Phillips et al., 2006). The large number of OSC enzymes has been characterized in plants with different substrate and product specificities (Thimmappa et al., 2014). These OSC enzymes catalyze the cyclization reactions of 2,3-oxidosqualene to form sterols, brassinosteroids, cucurbitacins, and scaffolds of other triterpenes (Yokota, 1997; Shang et al., 2014; Thimmappa et al., 2014). Those triterpene scaffolds were further modified by tailoring enzymes such as CYP450s, glycosyltransferases, methyltransferases and acyltransferases to produce all kinds of aglycones and glucosides of triterpenes (Thimmappa et al., 2014). Nomilin, the precursor and one of the major limonoids is a triterpenoid (Hasegawa et al., 1986; Ou et al., 1988; Manners, 2007). All kinds of limonoids are produced in the limonoids biosynthetic pathway (**Figure 1**) via oxidization, isomerization, methylation, acetylation and hydrolyzation of nomilin (Hasegawa et al., 1980; Hasegawa and Herman, 1985). Comparatively in recent decades, while many studies have focused on the how, when and where limonoids are biosynthesized and accumulated in citrus, there has been only a few on the genetic control of biosynthesis of limonoids. The discovery of non-bitter limonoid glucosides and identification of the limonoid glucosyltransferase gene (*LGT*) responsible for converting limonoid aglycones into corresponding non-bitter limonoid glucosides was the most important breakthrough in understanding naturally occurring limonoid debiting processing (Hasegawa et al., 1997). This provided the possibility to genetically modify the citrus to produce fruits free from limonoid bitterness which still could be beneficial to human health with high concentration of limonoid glucosides. However, improvement of overall production of limonoids is still not possible due to the key genes controlling the biosynthesis of limonoids have not yet to be identified.

Therefore, the aim of this study is to analyze the correlation of gene expression and limonoids contents with different citrus samples, from multiple genotypes, tissues and developmental stages to identify the factors that contribute to control of biosynthesis of limonoids.

## Materials and Methods

### Plant Materials and Sampling

All the citrus varieties used in this study were provided by the National Citrus Germplasm Repository, located in the Citrus Research Institute of Chinese Academy of Agricultural Sciences (CRIC), in Beibei, Chongqing, China.

For DGE analysis, leaf, phloem and seed samples were collected at same time from Dongfengzao pummelo (*C. grandis* (L.) Osbeck) on August, 2014. The leaf samples at three developmental stages (LI, LII, LIII) (Supplementary Figure S1) were collected. Phloem section in the internode between two leaves at the LII stage was collected and termed as PII. The collected seeds sample at this time was termed as SII. Those same samples were used for both transcriptome sequencing and determination of limonoids content.

For verification of important candidate genes involved in biosynthesis of limonoids, the seeds at four developmental stages were collected from fruits of the higher limonoids content variety Guangxi Shatian You (GXSTY) pummelo, and the lower limonoids content variety Early Siam pummelo as reported by Wang et al. (2016). Leaves were collected from seven citrus varieties which are significant different on limonoids contents, including Huapi Jingan (HPJG; *Fortunella crassifolia* Swingle), Rongan Jingan (RAJG; *F. crassifolia* Swingle), Gongchuan Migan (GCMG; *C. reticulata* Blanco), Wangcang Zoupi Gan (WCZPG; *C. reticulata* Blanco), GXSTY, Danna Xiangyuan (DNXY; *C. medica* L.), Beibei 447 Jingcheng (JC; *C. sinensis* (L.) Osbeck). All of the samples were immediately frozen in liquid nitrogen and stored at -80°C after collection and used for extraction of RNA and analysis of limonoids contents.

The seeds of GXSTY were germinated for virus-induced gene silencing (VIGS).

### RNA Extraction and Sequencing

Total RNA was extracted following the protocol provided by RNAprep pure Plant Kit (Tiangen Biotech (Beijing) Co., LTD., China). Transcriptome sequencing was conducted by BGI Tech Solutions Co., Ltd., Wuhan, China.

### Raw Data Processing, Functional Annotation, and Statistical Analyses

The raw reads were processed to remove the adapter sequences and low-quality reads (Length < 30 bp, quality < 9). The data have been submitted to NCBI database (NCBI accessions: SRP071684). Sequencing saturation analysis shows that the number of mapped genes tends to saturation (Supplementary Figure S2). The resulted high quality reads (Length ≥ 30 bp, high quality ≥ 9) were then mapped to the reference genome and gene sequences of *Citrus clementina* (Phytozome version 10.0) using TMAP software. The mapped reads were normalized by calculating the reads *per* kilobase *per* million mapped reads (RPKM) for each gene. Subsequently the *p* values and log_2_ of each gene were then calculated. Those genes were blasted to NCBI Non-redundant database (*E*-value ≤ 1e^-5^), and annotated with Blast2GO software^1^ and Kyoto Encyclopedia of Genes and Genomes database (KEGG) using BLASTx (*E*-value ≤ 1e^-5^). The differentially expressed genes (DEGs) were identified at threshold of | log_2_|≥ 1 and FDR < 0.001 based on the method of Poisson distribution (Audic and Claverie, 1997). Localization of DEGs into metabolic pathways was performed using the MapMan software (Thimm et al., 2004).

Particular expression patterns of differential expressed genes were analyzed with K-means clustering based on their log_2_ ratio. Visualizations of the clusters of different expression patterns were performed using MultiExperiment Viewer (MEV; version 4.9).

Amino acid sequences of *OSC* and CYP450s genes were downloaded from NCBI database^2^ following published reports (Thimmappa et al., 2014). Sequences alignment and phylogenetic analysis were performed with MEGA5.0 software. Phylogenetic trees were constructed using the neighbor-joining method with 1,000 bootstrap replicates. The accessions of *OSC* and CYP450s genes are listed in Supplementary Files S1, S2, respectively.

### Quantitative Real-Time PCR (qRT-PCR) Analyses

Some highly differential expressed transcripts identified by DGE were selected as candidate genes for qRT-PCR analysis. The leaves of seven varieties and the seeds of GXSTY and ESP from 4 different developmental stages were used to validate the expression of those candidate genes in relation to the biosynthesis of limonoids. The primers of qRT-PCR were designed using Primer premier 5.0 software (Supplementary Table S1).

1.0 μg total RNA was reversely transcribed into single strand cDNA using RevertAid First Strand cDNA Synthesis Kit (Thermo Scientific, USA). qRT-PCR reaction was performed in a total volume of 20 μl. It contained 1 × iTaq^TM^ Universal SYBR^®^ Green Supermix (Bio-Rad, USA), 200 nM of each primer, and 2 μl cDNA template (ten-fold dilution of first strand cDNA). The PCR program was performed as follows: initial denaturation for 10 min at 95°C, 40 cycles of 15 s at 95°C and 60 s at 59°C on a Real-time PCR system (Applied Biosystems 7500 Fast, Applied Biosystems, USA). “No template controls” (NTC) were included in each run and each sample was set in three technical replicates. The citrus *Actin* and *GAPDH* genes were used as the endogenous reference genes to normalize the expression level of the genes. Relative quantity of the expression of each gene was calculated using the 2^-ΔΔCt^ method (Livak and Schmittgen, 2001).

### Silencing of *CiOSC* Gene with VIGS

A 367 bp fragment of *CiOSC* amplified from the RNA of leaves of GXSTY with *CiOSC*-V2 primers (Supplementary Table S1) was inserted into *Xba*I and *Bam*HI sites of the TRV vector (TRV-derived vector was supplied by Prof. Yuncong YAO from Beijing University of Agriculture). The constructions of TRV1 and TRV2 were transformed into *Agrobacterium tumefaciens* strain EHA105 by electroporation. *Agrobacterium* suspensions of TRV1 and TRV2 were prepared as previously described (Liu et al., 2002). Germinated seeds from GXSTY variety were submerged into *Agrobacterium* suspensions inside of a vacuum chamber as previously reported (Deng et al., 2012; Yan et al., 2012). After vacuum infiltration, the germinated seeds were rinsed with water, sown in soil in a growth chamber. Thirty days post-infiltration (dpi), the whole plants were ground into fine powder in liquid nitrogen immediately after removal of young leaves and shoot apex, and stored at -80°C.

The extraction of total RNA and cDNA synthesis were performed using the method described above. The expression of *CiOSC* gene was determined with qRT-PCR analysis. Plants infected with empty vector, and non-infected plants were used as controls. To normalize the gene expression, *CiGAPC2* was used as a reference gene. All the samples were set in three technical replicates.

### Determination of Limonoids Contents

The contents of limonin and nomilin were determined by HPLC in two replicates as reported previously (Wang et al., 2016). Nomilin and limonin were extracted from the samples twice with dichloromethane in an ultrasonic bath for 20 min per extraction. The ratio of dichloromethane (mL) to sample (grams) was 10: 1. For HPLC analysis, the column was maintained at 30°C. Fifteen microliter sample solutions were injected to run in a mobile phase consisting of acetonitrile: deionized water at 40: 60 (V:V) with a flow rate of 1 ml/min. Nomilin and limonin were detected at 210 nm wavelength. The total content of limonoids is the sum of the contents of limonin and nomilin.

## Results

### Digital Gene Expression Analyses

A total 14,551,995 (LI), 13,302,783 (LII), 14,900,160 (LIII), 18,151,136 (PII, stage 2 phloem) and 14,978,415 (SII, stage 2 seeds) clean reads were obtained (**Table 1**) after raw reads processing. About 89.05–98.99% of clean reads were mapped to the reference genome (**Table 1**). The number of mapped genes was 23,477 in LI, 23,608 in LII, 23,495 in LIII, 24,323 in PII, and 21,789 in SII. Those genes were blasted with public databases of Nr, GO and KEGG.

**Table 1:** Summary of statistics of digital gene expression profiling sequencing data and detail of the mapping clean reads against *Citrus clementina* reference genome.

	Total clean reads	Total base pairs	Total mapped reads	Unique matched	Multi-position matched	Total unmapped reads
LI	14,551,995	2,088,362,571	4,404,512 (98.99%)	13,819,989 (94.97%)	584,523 (4.02%)	147,483 (1.01%)
LII	13,302,783	1,878,765,769	2,995,811 (97.69%)	12,514,069 (94.07%)	481,742 (3.62%)	306,972 (2.31%)
LIII	14,900,160	2,020,104,150	14,601,632 (98.00%)	14,070,039 (94.43%)	531,593 (3.57%)	298,528 (2.00%)
PII	18,151,136	2,564,539,535	17,382,416 (95.76%)	16,730,873 (92.18%)	651,543 (3.59%)	768,720 (4.24%)
SII	14,978,415	2,091,508,996	13,338,996 (89.05%)	12,975,619 (86.63%)	363,377 (2.43%)	1,639,419 (10.95%)

*^∗^Length cutoff ≥ 30 bp; High-quality ≥ 9.*

**FIGURE 2:**
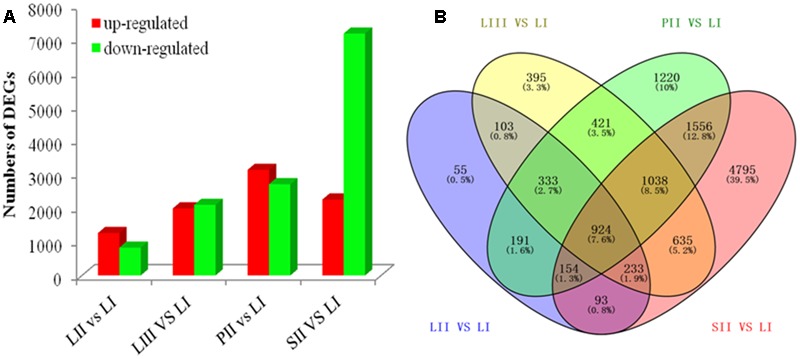
**The number of genes differentially expressed among different tissues. (A)** Up- and down-regulated genes in different groups. **(B)** Venn diagram of the number of genes differentially expressed in different groups.

Differential expression analysis was performed by pair-wise comparison using |log_2_|≥ 1 and FDR < 0.001 as thresholds. LI was defined as the reference sample because of its low limonoids content. As shown in **Figure 2A**, the numbers of DEGs were 2,086 between LII and LI, 4,082 between LIII and LI, 5,837 between PII and LI, and 9,428 between SII and LI. Venn Diagram analysis indicated that 924 DEGs were shared across all four sets of paring comparisons, which accounted for 7.6% of the total genes (**Figure 2B**). However, only 47 out of 924 genes were annotated to be involved in secondary metabolism.

**FIGURE 3:**
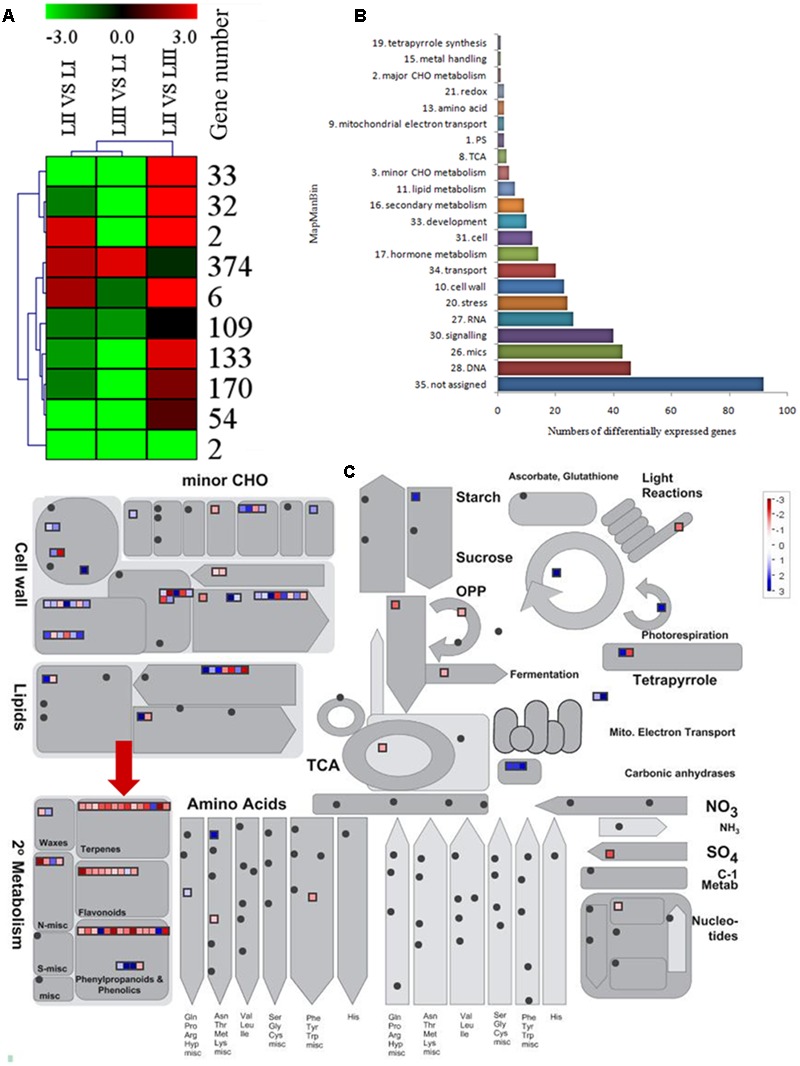
**Selection of differentially expressed genes. (A)** K-means cluster of 924 DGEs. **(B)** MapMan functional annotations of 382 genes. **(C)** MapMan analysis of genes participated in metabolism. Red arrow shows genes belong to terpene biosynthesis.

To search for the genes involved in the biosynthesis of limonoids, K-means cluster analysis was performed using the genes expressed in leaves at three stages to investigate their expression profiles. The 924 shared DEGs were clustered into ten groups. Genes with the same expression pattern were filtered by MEV software (**Figure 3A**). More than two-third of genes were characterized to 22 functional categories such as DNA, mics, signaling, RNA, stress, cell wall, transport, hormone metabolism, cell, development, secondary metabolism and lipid metabolism. There were 92 genes not assigned to any functional categories (**Figure 3B**).

### Functional Classification of Differentially Expressed Genes

Limonoids are synthesized from the triterpenoids structure precursors through MEP and MVA pathways (Cheng et al., 2007). There were 14 genes related to biosynthesis of triterpenoids among the 924 DEGs (**Figure 3C**), which have not been reported except the *LGT* gene. A method combining functional classification and K-means cluster was used to filter the genes participating in biosynthesis of limonoids. *OSC, CYP450s, TFs, UGTs* and acyltransferase were highlighted based on functional classifications of GO and KEGG. Those genes included *OSC* (ciclev10010416m), *CYP450s* (ciclev10031193m, ciclev10030448m, ciclev10030225m, ciclev10000884m, ciclev10004733m, ciclev10019616m, ciclev10004103m, ciclev10011511m, ciclev10031272m), *TFs* (ciclev10021695m, ciclev10005095m, ciclev10011386m, ciclev10012453m), *UGTs* (ciclev10020010m, ciclev10015042m, ciclev10001658m) and one acyltransferase (ciclev10001521m) (**Table 2**). By analyzing expression patterns of K-means clustering, three conjunctive clusters (including 2, 6, and 374 genes, respectively) were found to be possibly correlated with biosynthesis of limonoids. The three clusters contained 382 genes accounting for 41.3% of shared DEGs.

**Table 2:** Candidate genes related to biosynthesis of limonoids.

Gene	Enzyme	KO id (EC: No)	GO
ciclev10010416m	Oxidosqualene cyclase	K01853(EC:5.4.99.8)	GO:0016871
ciclev10021695m	MYB domain transcription factor family	K09422	GO:0003677
ciclev10011386m	WRKY domain transcription factor family	K13424	–
ciclev10005095m	WRKY domain transcription factor family	K13425	GO:0005488
ciclev10012453m	Basic Helix-Loop-Helix family	K11253	–
ciclev10031065m	CYP714A1	K10717	GO:0020037
ciclev10030087m	CYP89A6	K00517(EC:1.14.–.–)	GO:0020037
ciclev10022473m	CYP71B37	K11818(EC:1.14.–.–)	–
ciclev10000886m	CYP83B1	K11818(EC:1.14.–.–)	GO:0046872
ciclev10031272m	CYP89A5	K00517(EC:1.14.–.–)	GO:0020037
ciclev10025448m	CYP90D1	K12638(EC:1.14.13.112)	GO:0020037
ciclev10001521m	Acyltransferases	K13510(EC:2.3.1.23 2.3.1.67)	GO:0016746
ciclev10020010m	UDP glucosyltransferases	K08237(EC:2.4.1.218)	GO:0035251
ciclev10015042m	UDP glucosyltransferases	K08237(EC:2.4.1.218)	GO:0035251
ciclev10001658m	UDP glucosyltransferases	K13648(EC:2.4.1.43)	GO:0016757

**FIGURE 4:**
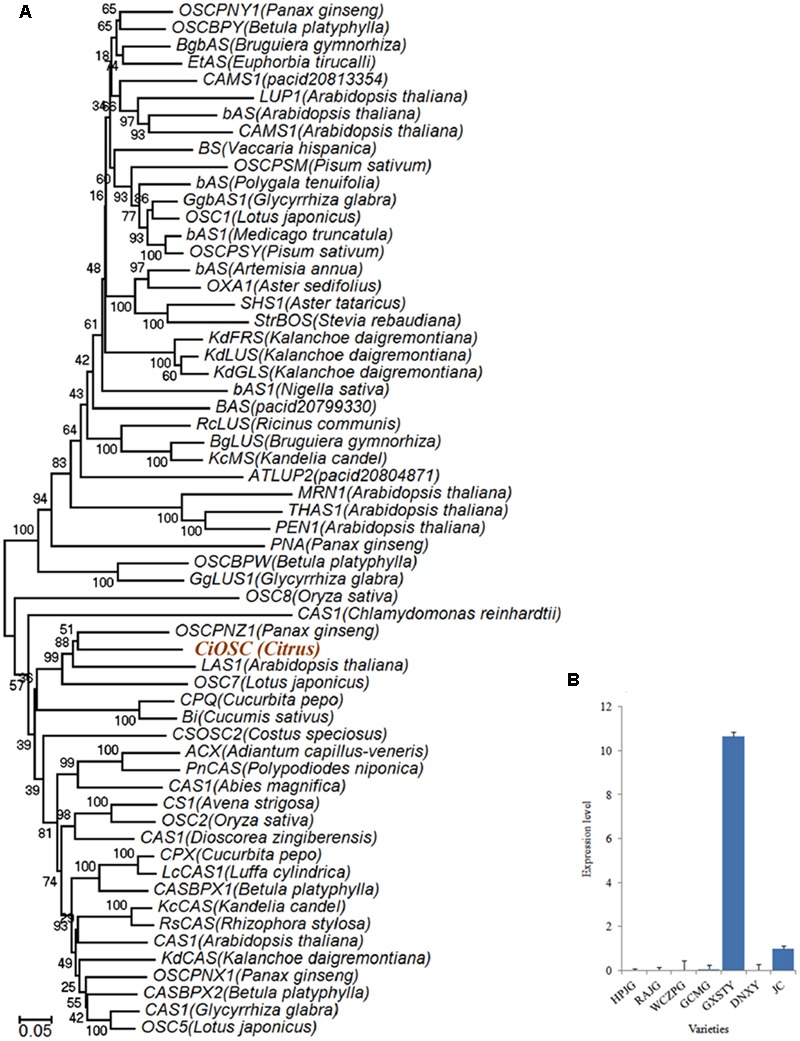
**Phylogenetic tree and expression level of *CiOSC* genes. (A)** Phylogenetic tree of plant oxidosqualene cyclase family members (gene accessions provided in Supplementary File S1). Nodes are labeled with the percentage of bootstrap iterations. *CiOSC* is clustered in the same sub-clade with *Bi* and *CPQ*. **(B)** qRT-PCR analysis of *CiOSC* among different varieties. HPJG, Huapi Jingan; RAJG, Rongan Jingan; WCZPG, Wangcang Zoupi Gan; GCMG, Gongchuan Migan; GXSTY, Guangxi Shatian You; DNXY, Danna Xiangyuan; JC, Jincheng.

Functional analysis indicated that in the 382 genes, 7 of them participate in secondary metabolisms of phenylpropanoids, alkaloid, wax, phenols, terpenoids and flavonoids. ciclev10010416m (termed as *CiOSC*) was the only gene involved in the biosynthesis of triterpenoids. The *OSC* gene encodes OSC, a key enzyme in terpenoids biosynthetic pathway. Phylogenetic analysis demonstrated that *CiOSC* was clustered in the same sub-clade with cucurbitadienol synthases genes *CPQ* and *Bi*, which regulate biosynthesis of triterpenoids in squash (*Cucurbita pepo*) and cucumber (*Cucumis sativus* L.), respectively (Shibuya et al., 2004; Shang et al., 2014; **Figure 4A**). Therefore, *CiOSC* could be one of the key genes affecting biosynthesis of limonoids through regulating the production of triterpenoids.

Sixty-six transcription factor genes (*TFs*) were present among the 924 DEGs, covering 19 families. The *AP2/EREBP* and *bHLH* are the two largest families. In this study, only six *AP2/EREBP* members and one *bHLH* member were positively correlated with limonoids contents in the leaf samples LII and LIII. The expression of the remaining *TFs* (*WRKY, C2H2, MYB, Trihelix*, and *GRAS* members) also corresponded to limonoids contents.

Among 382 genes related to biosynthesis of limonoids, 13 of them (ciclev10031193m, ciclev10030448m, ciclev10030225m, ciclev10000884m, ciclev10004733m, ciclev10019616m, ciclev10004103m, ciclev10011511m, ciclev10031272m, ciclev10030087m, ciclev10022473m, ciclev10000886m, ciclev10031065m) were annotated as *CYP450*.

Three genes (ciclev10020010m, ciclev10015042m, ciclev10001658m) were identified as *UGTs*. Their expression patterns were correlated with the limonoids contents at three development stages of the leaves. Two of them (ciclev10020010m and ciclev10015042m) belonged to *UGT72* families.

### Correlation Analyses of Candidate Genes

To verify the DEGs obtained from sequencing and the bioinformatic analyses were indeed corresponding to the gene expression profiles, 20 candidate genes correlated with biosynthesis of limonoids were selected for qRT-PCR analysis. The results demonstrated that the expression profiles were consistent between the results of DGE and qRT-PCR (Supplementary Figure S3).

The materials of developing leaves, phloem and seeds from Dongfengzao pummelo, and the seeds at four development stages from ESP and GXSTY varieties were used for validating the genes correlated with biosynthesis of limonoids (**Table 3**). The expression patterns of 15 genes were found to correspond to the levels of limonoids contents. The results of Poisson correlation analysis showed that the expression levels of most of the genes were positively correlated with limonoids contents in ESP and GXSTY (**Table 4**). Correlation coefficient of *CiOSC* between gene expression levels and limonoids contents in ESP and GXSTY were at 0.722 and 0.741, but not high enough levels to be significant. The expression levels of *CiOSC* were significantly different among the varieties tested, and highly linked to the limonoids contents. High levels of expression were found in GXSTY and JC, which had higher limonoids contents. The reverse was observed in the lower limonoids content varieties of HPJG, RAJG, WCZPG, GCMG, and DNXY (**Figure 4B**).

**Table 3:** Limonoids contents in different tissues^∗^.

Varieties	Tissues	Limonin	Nomilin	Limonoids
Dongfengzao pummelo	LI	20.14 ± 0.40	0.00	20.14
	LII	46.45 ± 2.86	27.67 ± 0.22	74.12
	LIII	21.98 ± 1.54	0.00	21.98
	PII	126.11 ± 6.51	18.20 ± 3.35	144.31
	SII	171.77 ± 9.40	236.71 ± 12.66	408.48
Early Siam Pummelo	ST1	135.25 ± 2.53	126.20 ± 1.73	261.45
	ST2	1168.09 ± 12.59	1104.56 ± 5.10	2272.64
	ST3	1190.68 ± 22.85	1639.48 ± 25.57	2830.16
	ST4	1982.07 ± 29.38	2108.34 ± 28.73	4090.41
Guangxi Shatian You	ST1	30.84 ± 3.39	40.96 ± 4.62	80.38
	ST2	583.37 ± 3.43	1337.68 ± 3.91	1920.84
	ST3	1423.39 ± 5.00	2482.98 ± 2.66	3906.37
	ST4	1006.66 ± 6.06	1560.98 ± 9.45	2567.64

*^∗^Mean ± SD (standard deviation; *n* = 3). Unit: mg.kg^-*1*^ FW.*

**Table 4:** Relationship between expression levels of candidate gene and limonoids contents during seed development.

	A	B	C	D	E	F	G	H	I	J	K	L	M	N	O
ESP	0.722	0.837	0.858	0.822	0.884	0.695	0.985^b^	0.865	0.921	0.938	0.948	0.554	-0.754	-0.612	-0.851
GXSTY	0.741	0.976^b^	0.812	0.765	0.898	0.317	0.92	0.958^b^	0.995^a^	0.902	0.981^b^	0.876	0.771	0.736	0.652

*ESP, Early Siam Pummelo; GXSTY, Guangxi Shatian You. (A), ciclev10010416m (*CiOSC*); (B), ciclev10021695m (*MYB*); (C), ciclev10011386m (*WRKY*); (D), ciclev10005095m (*WRKY*); (E), ciclev10012453m (*bHLH*); (F), ciclev10031065m (*CYP450*); (G), ciclev10030087m (*CYP450*); (H), ciclev10022473m (*CYP450*); (I), ciclev10000886m (*CYP450*); (J), ciclev10031272m (*CYP450*); (K), ciclev10025448m (*CYP450*); (L), ciclev10001521m (acyltransferases); (M), ciclev10020010m (*UGT*); (N), ciclev10015042m (*UGT*); (O), ciclev10001658m (*UGT*).*

This verification was carried out with the remaining 14 genes, including four *TFs*, six *CYP450s*, three *UGTs* and one acyltransferase genes, which had the same expression patterns in developing leaves. The amino acid sequences of CYP450s and UGTs are included in the Supplementary Files S2, S3. Phylogenetic analysis classified six *CYP450s* into three clusters (Supplementary Figure S4).

The expression of the candidate genes demonstrated close relationship with the limonoids contents during seed development. In particular, significantly positive correlations were observed between the levels of gene expression and the limonoids contents in ciclev10021695m (*MYB*), ciclev10030087m (*CYP450*), ciclev10022473m (*CYP450*), ciclev10000886m (*CYP450*), and ciclev10025448m (*CYP450*) genes, with the correlation coefficients between 0.958^b^ and 0.995^a^. The expression of three *UGTs* genes (ciclev10020010m, ciclev10015042m, ciclev10001658m) showed different correlations with limonoids contents in the developing seeds of ESP and GXSTY. In low limonoids content variety GXSTY, *UGTs* genes expressed positively (correlation coefficients 0.652∼0.771) with limonoids contents during seeds development, but negative correlations (-0.61 ∼-0.85) with limonoids contents in high limonoids content variety ESP.

### Confirmation of *CiOSC* Gene Related to the Biosynthesis of Limonoids

**FIGURE 5:**
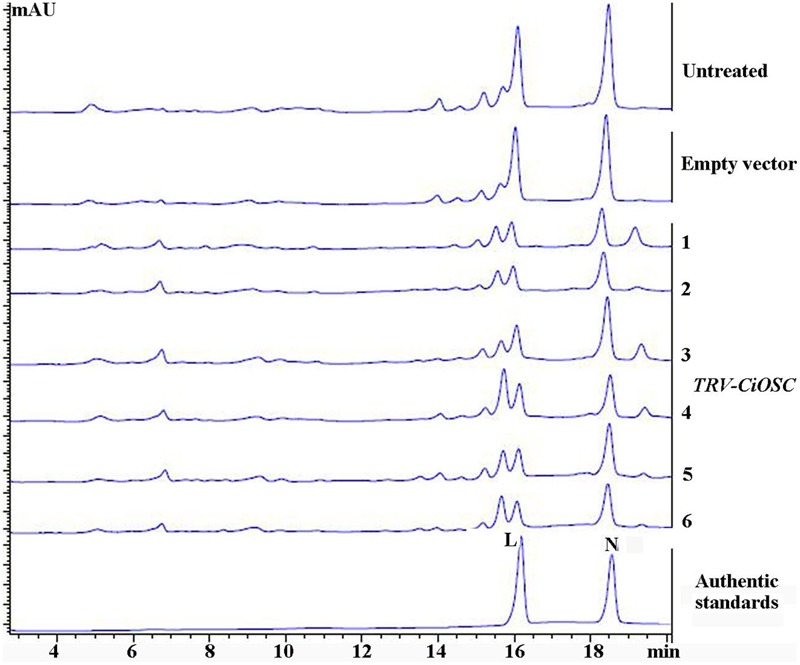
**The contents of limonin and nomilin in CiOSC silenced seedlings analyzed by HPLC.** L, limonin; N, nomilin.

Virus-induced gene silencing was used to silence the expression of the *CiOSC* gene in the seedlings of *C. grandis* variety GXSTY. Six positive seedlings with significantly reduced expression of *CiOSC* gene were obtained from ten infiltrated seedlings (**Table 5** and Supplementary Figure S5). No obvious morphological differences were observed among untreated seedlings, seedlings infected with empty vector, and TRV-*CiOSC* vector (Supplementary Figure S6). Compared to the seedlings infiltrated with empty vector, the level of gene expression in those six seedlings decreased to 9.09∼51.10% (**Table 5**). Consequently, the limonin and nomilin contents of those seedlings were reduced to 35.08∼50.59% and 44.49∼60.83%, respectively (**Table 5** and **Figure 5**). The relative expression level of *CiOSC* gene in one of the transgenic plants was significantly inhibited (only 9.09% of control). Consequently contents of limonin and nomilin were reduced to only 35.08 and 44.49% of the control, respectively.

**Table 5:** Relative gene expression, limonin and nomilin contents in *CiOSC* silenced plants.

Samples	Relative content of limonin (%)	Relative content of nomilin (%)	Relative expression level of *CiOSC* gene (%)
1	39.00	47.81	14.02
2	35.08	44.49	9.09
3	47.15	75.85	22.29
4	50.59	50.17	25.30
5	40.97	60.83	42.85
6	39.54	47.61	51.10
Empty vector	100.00	100.00	100.00
Untreated	107.90	101.39	103.04

## Discussion

It is generally known that triterpenes are the primary precursor for synthesis of limonoids. Some of the *CYP450s* have been reported to play critical roles in modification of triterpenes, including hydroxyl, ketone, aldehyde, carboxyl, or introduction of epoxy groups (Shang et al., 2014; Thimmappa et al., 2014). Seven out of nine genes controlling biosynthesis of cucurbitacin C, the bitterness substance in cucumber, were members of *CYP450s* (Shang et al., 2014). In this study, the *CYP450* family members of ciclev10030087m, ciclev10000886m, ciclev10031272m, ciclev10025448m, ciclev10031065m, and ciclev10022473m were found significantly corresponding to the accumulation of limonoids during the seeds development in ESP and GXSTY. This suggests that those genes are obviously involved in the biosynthesis of limonoids.

*UGTs* were the other key genes identified by DGE analysis. They are the essential genes regulating glucosylation of triterpenes. This study demonstrated positive correlation with limonoids contents, in low limonoids content pummelo genotype but negative correlation, in high limonoids content pummelo genotype through the gene expression analysis of *UGTs* (ciclev10020010m, ciclev10015042m and ciclev10001658m). This may be due to the difference in ratios of limonoid glucosides to nomilin and limonin between those two varieties, as the function of *UGTs* is to convert nomilin and limonin to limonoid glucosides. To prove this, further work on analysis of limonoid glucosides contents in these two varieties will be required. However, it has been reported that in some limonoids rich varieties, most of limonoids accumulated as nomilin and limonin, instead of limonoid glucosides (Kita et al., 2003; Zaare-Nahandi et al., 2008).

**FIGURE 6:**
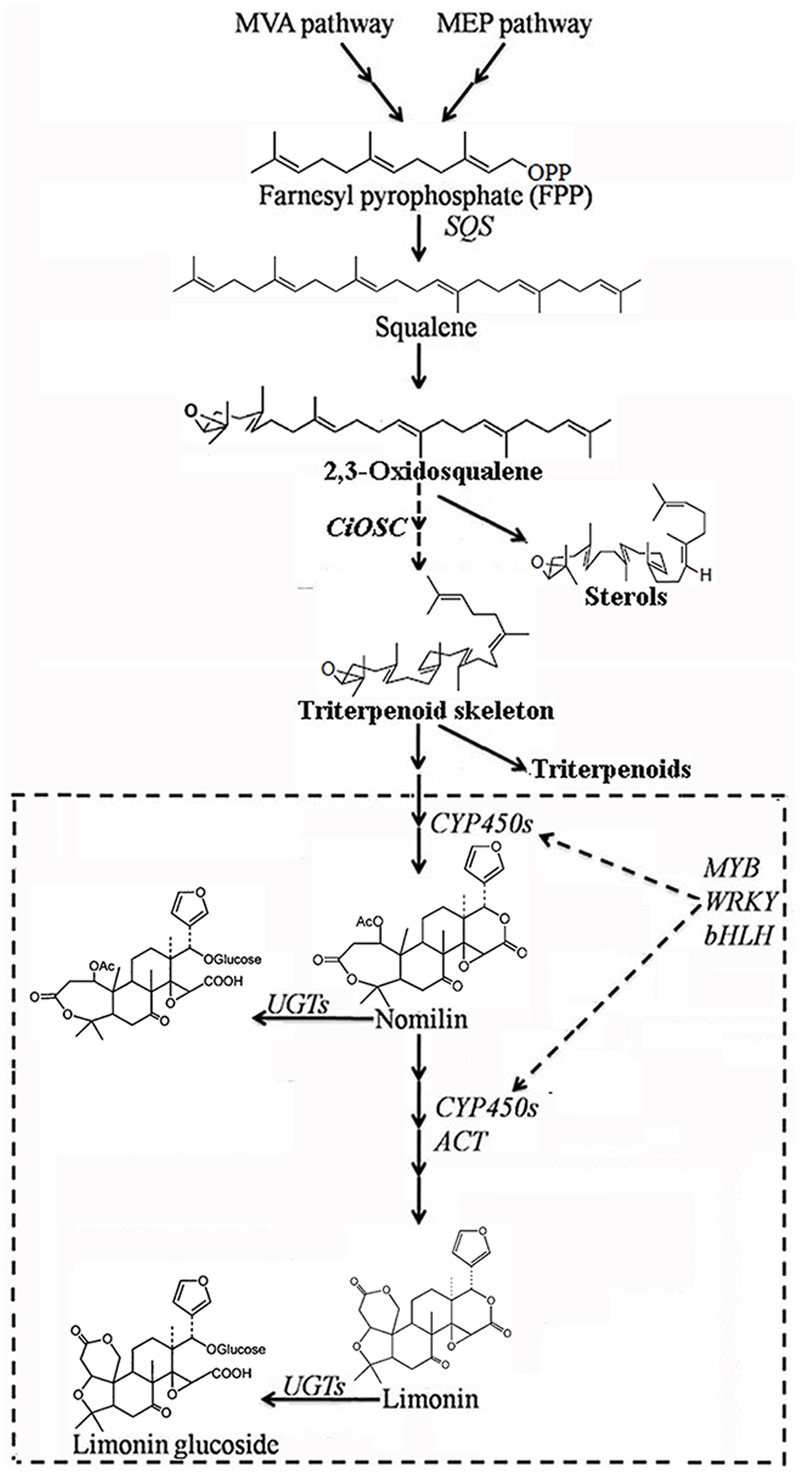
**The proposed pathway of limonoids biosynthesis in citrus.** *UGTs*, UDP glucosyltransferases; *ACT*, acyltransferase; *OSC*, oxidosqualene cyclase; *CYP450s*, cytochrome P450s; *SQS*, Squalene synthase.

Oxidosqualene cyclases are critical enzymes for converting 2,3-oxidosqualene or squalene into the precursor of triterpene scaffolds (Thimmappa et al., 2014). Limonoids is one group of triterpenoids specially synthesized via MVA and MEP pathways (Ou et al., 1988; Endo et al., 2002). However, to date, there has been no report on involvement of *OSC* genes in biosynthesis of limonoids. In the present study, DGE analysis indicated that the expression of *CiOSC*, a member of *OSC* genes in pummelo corresponded to the biosynthesis of limonoids. Further investigation demonstrated that the expression of *CiOSC* was highly correlated to the limonoids contents among pummelo varieties. Suppression of *CiOSC* expression with TRV-mediated VIGS resulted in the reduced expression of *CiOSC* which led to the significantly decreased production of limonoids. The results suggested that *CiOSC* could be a critical gene participating in biosynthesis of limonoids in citrus. The evidence also showed that the expression of *CiOSC* was much higher in the leaves and phloem than that of the seeds. Previous studies reported that nomilin is synthesized in the phloem, and transported to the other organs and tissues to synthesize into different forms of limonoids (Hasegawa and Hoagland, 1977; Ou et al., 1988). *OSCs* are responsible for the synthesis of the pre-structure of triterpenoids in many plant species. The *CiOSC* should act through controlling the production of triterpenoids to regulate the biosynthesis of nomilin in citrus. Phylogenetic analysis revealed that *CiOSC* was the ortholog of *Bi* and *CPQ* genes, which were the first committed enzymes of cucurbitacin biosynthesis in cucumber and in squash, respectively (Shibuya et al., 2004; Shang et al., 2014). The significant involvement of *CiOSC* in biosynthesis of limonoids demonstrated in this study suggests that a large portion of triterpenoids synthesized by *CiOSC* may be supplied to the biosynthesis of limonoids in citrus. Based on the genes related to the biosynthesis of limonoids identified in this study, a preliminary draft of genetic factors in the limonoids biosynthetic pathway could be proposed (see **Figure 6**).

In citrus, limonoids consist of 37 limonoid aglycones and 17 limonoid glucosides (Alford and Murray, 2000; Chowdhury et al., 2003; Patil et al., 2009). Previous research reported that *TFs* could regulate multiple genes in the biosynthesis of indole alkaloids (Van Moerkercke et al., 2015), artemisinins (Yu et al., 2012; Zhang et al., 2015), anthocyanins (Butelli et al., 2012; Rinaldo et al., 2015) and flavonols (Misra et al., 2010; Stracke et al., 2010). This study concludes twenty-six *TFs* including *AP2, WRKY, bHLH*, and *MYBs* demonstrated certain relationships with the biosynthesis of limonoids. Further work is required to clarify their roles in the biosynthesis of limonoids, and uncovering the key genes involved in the regulation of biosynthesis of limonoids.

The first glimpse of information on genetic study of biosynthesis of limonoids needs further investigation and verification of the functions of those putative genes. Identification of the major genetic factors on the limonoids biosynthetic pathway should be possible with more research.

## Author Contributions

FW designed the study, performed experiment, ran data analysis and prepared the draft of manuscript. MW, XL, and YX performed some of experiments. SZ and WS participated in some of experiments and assisted in the preparation of manuscript. XZ is responsible for overall supervision of the study and revised subsequent versions of the manuscript. All authors have read and approved the final version of the manuscript.

## Conflict of Interest Statement

The authors declare that the research was conducted in the absence of any commercial or financial relationships that could be construed as a potential conflict of interest.

## Footnotes

1 http://www.geneontology.org/

2 http://www.ncbi.nlm.nih.gov/protein/?term
