# Supplementary material for: Identification of Putative Genes Involved in Limonoids Biosynthesis in Citrus by Comparative Transcriptomic Analysis
Source: Front Plant Sci. 2017 May 12;8:782. doi: 10.3389/fpls.2017.00782 (PMC5427120; doi:10.3389/fpls.2017.00782)
Supplement: Supplementary file 1 [file Data_Sheet_1.DOC]

Supplementary files


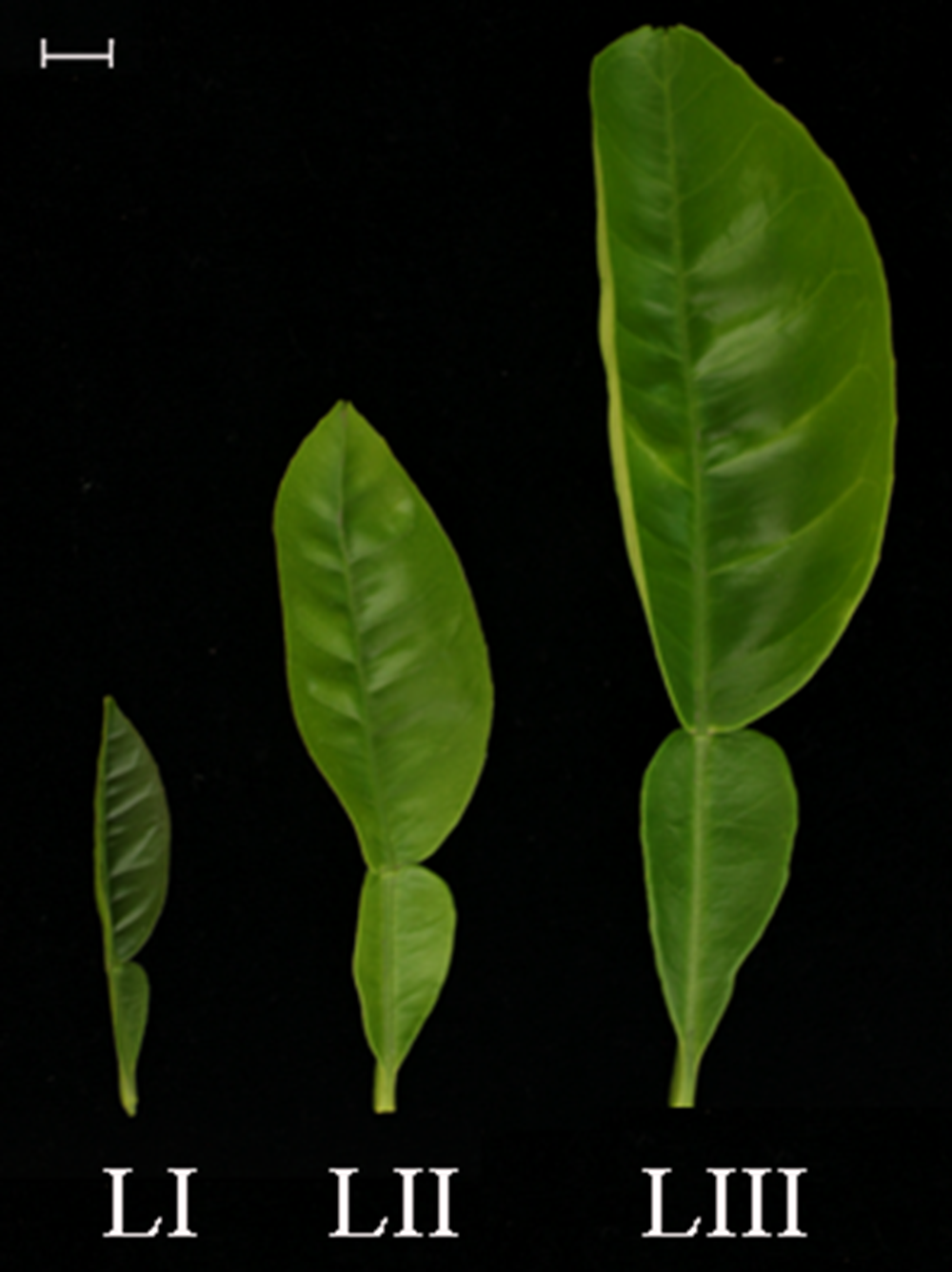


Fig. S1 Development stages of the leaves of Dongfengzao pummelo (*Citrus grandis* (L.) Osbeck)


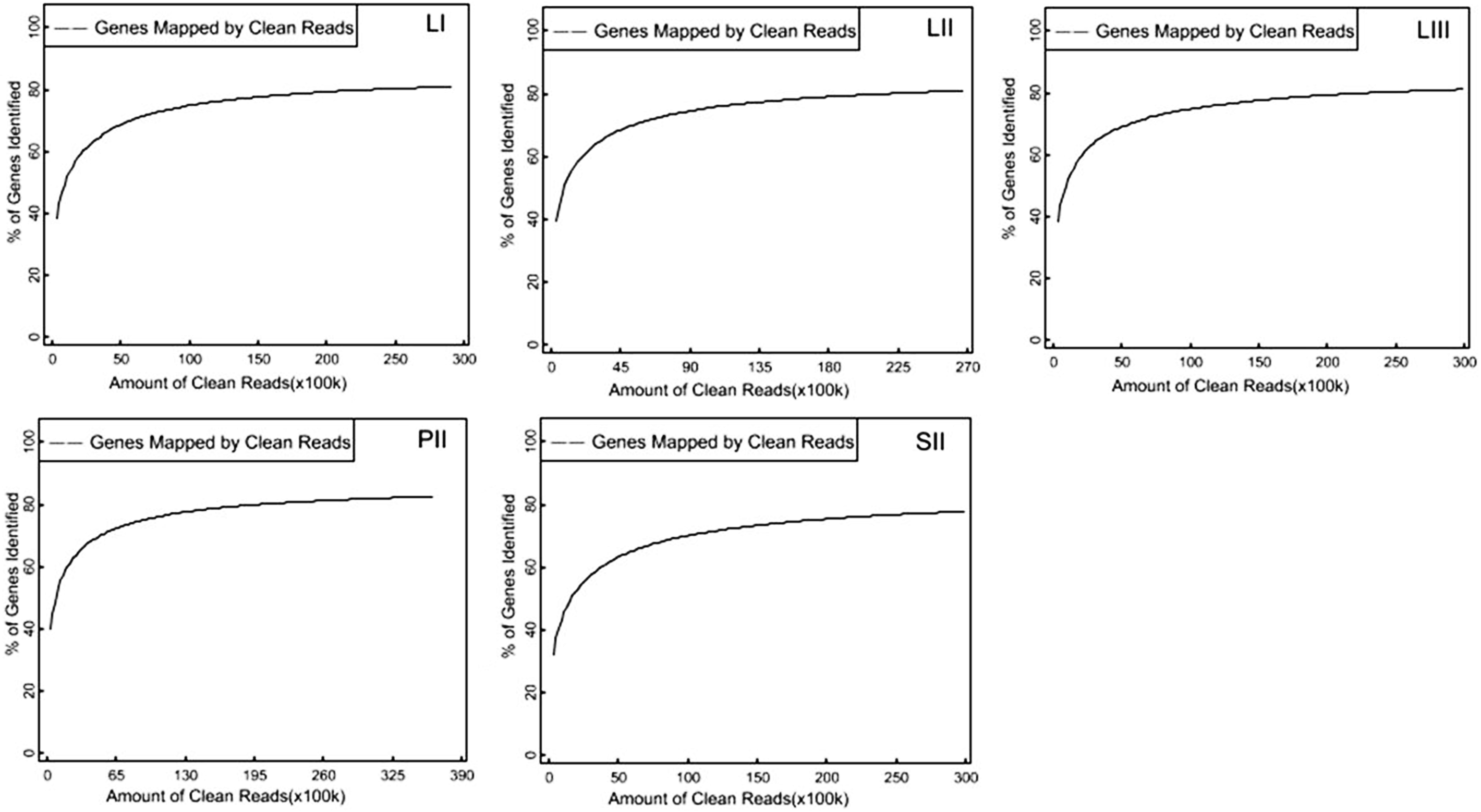


Fig. S2 Analysis of sequencing saturation in five tissues.


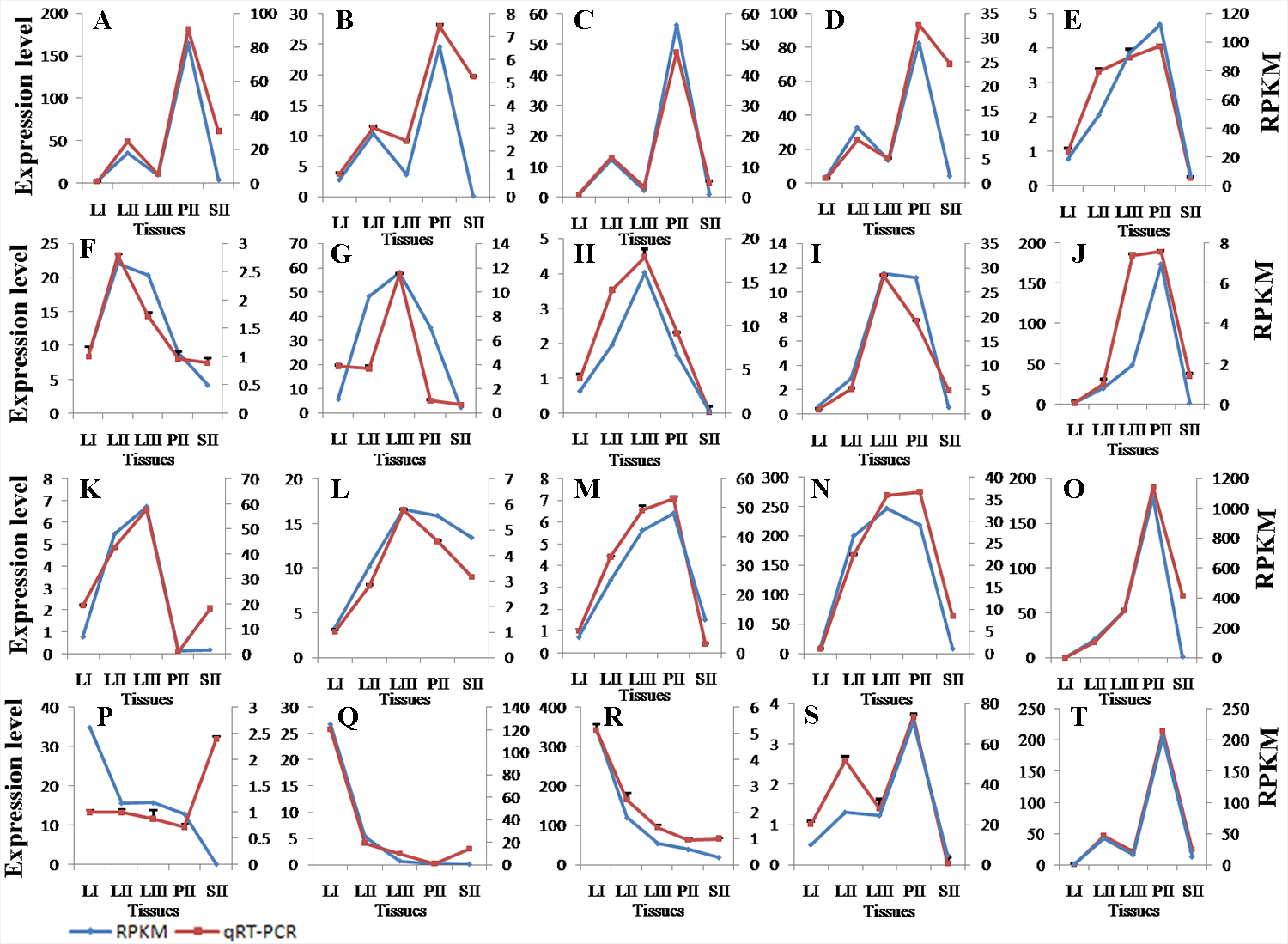


Fig. S3 Comparison of RPKM values of RNA-seq and qRT-PCR analysis.

(A), ciclev10008930m (WRKY); (B), ciclev10000895m (CYP450); (C), ciclev10009761m (WRKY); (D), ciclev10019989m (CYP450); (E), ciclev10001658m (UGT); (F), ciclev10030153m (bHLH); (G), ciclev10022473m (CYP450); (H), ciclev10015042m (UGT); (I), ciclev10010416m (OSC); (J), ciclev10025382m (CYP450); (K), ciclev10025448m (CYP450); (L), ciclev10031272m (CYP450); (M), ciclev10020010m (UGT); (N), ciclev10031286m (CYP450); (O), ciclev10031134m (phenols synthase); (P), ciclev10015582m (phytoene synthase); (Q), ciclev10015700m (ethylene metabolism); (R), ciclev10012786m (gibberellin metabolism); (S), ciclev10031193m (CYP450); (T), Ciclev10005570m (AP2/EREBP).


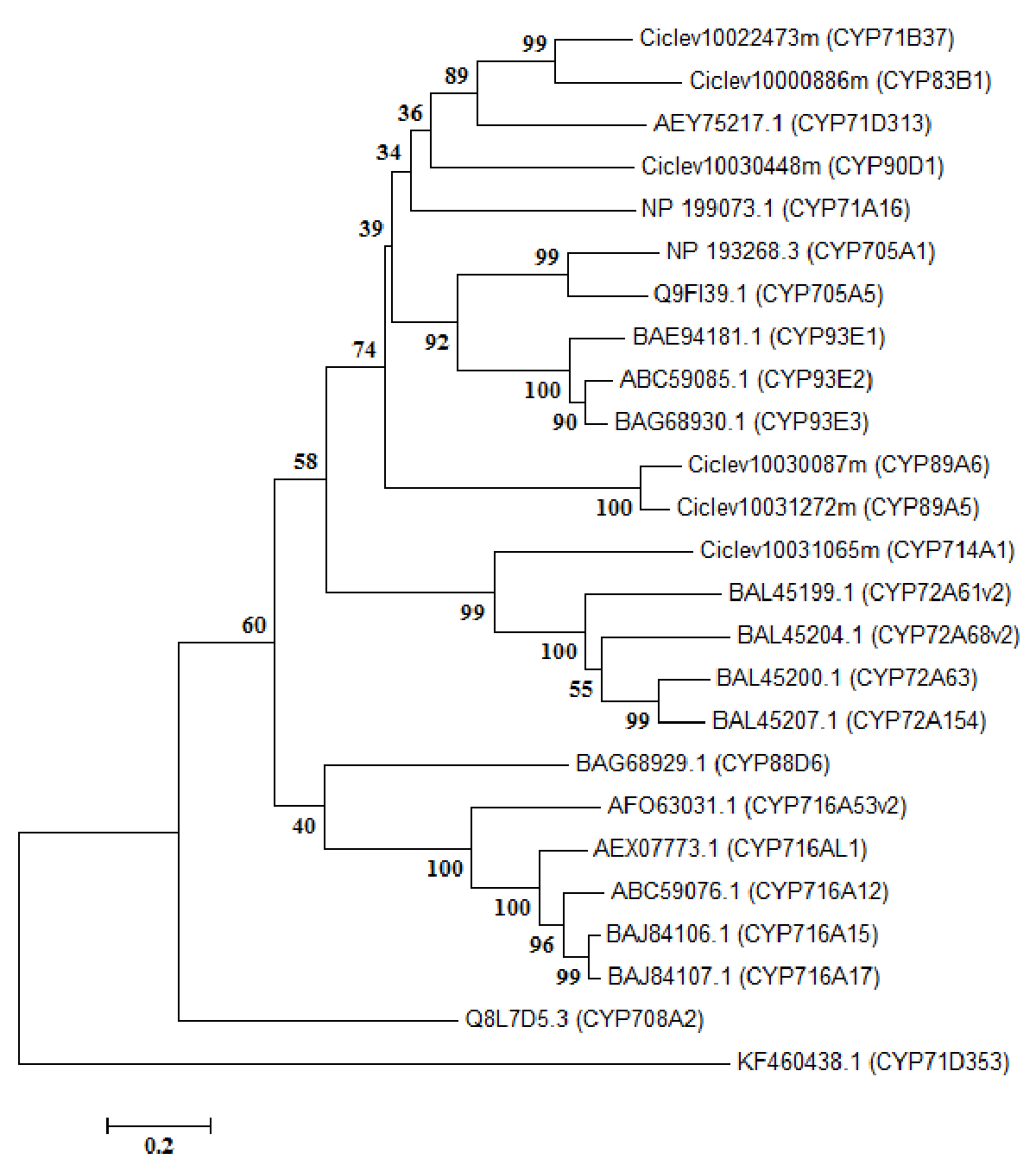
Fig. S4 Phylogenetic tree of triterpene-modifying cytochrome P450s family members in plants.


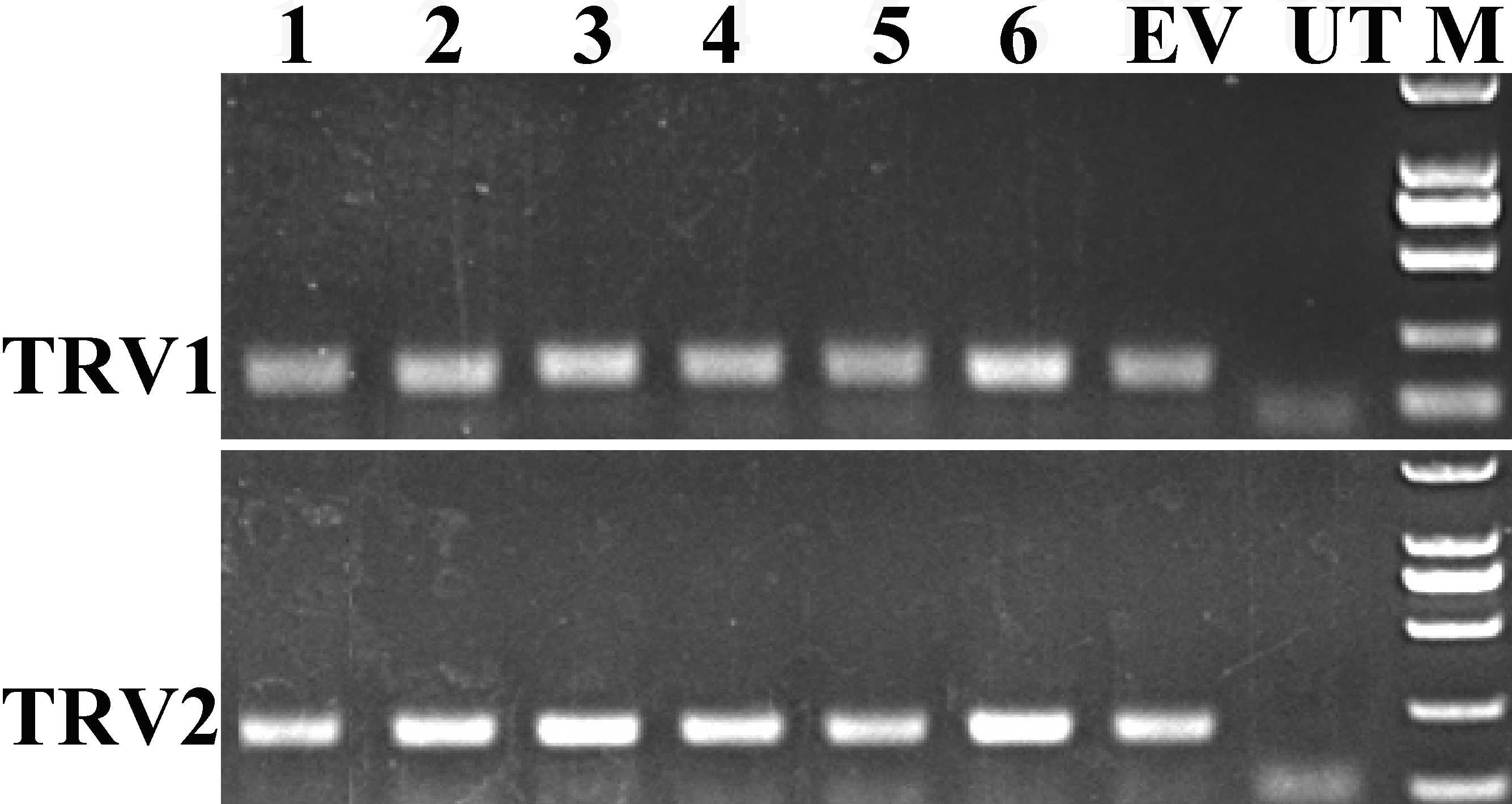


Fig. S5 PCR results of *CiOSC* gene silenced seedlings.

UT, untreated; EV, empty vector; M, DL2000 marker.


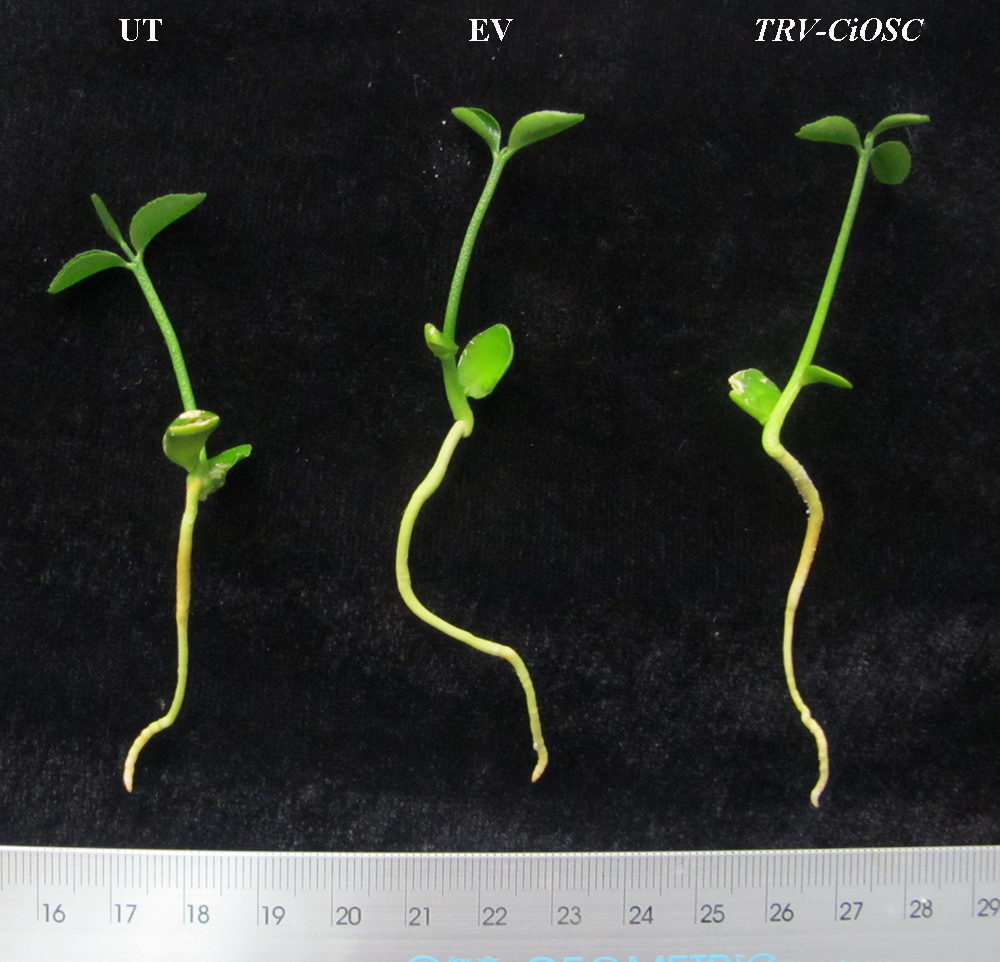


Fig. S6 Morphology of *CiOSC* gene silenced seedlings.

UT, untreated; EV, empty vector.

Table S1 Primers used for qRT-PCR validation.

| Gene ID | Forward (5'-3') | Reverse (5'-3') | Tm (℃) |
| --- | --- | --- | --- |
| ciclev10010416m | CTTTTGGCCTGGTGACTATGGT | CATTTCCCTTCGATGTTCCACT | 59 |
| ciclev10021695m | GGGCGAACCGATAACGAGAT | GCTGGAGTCTGGCAGCAAAT | 58 |
| ciclev10011386m | GCTGCAGAGCACAGGGAGTT | TAAGTCATCCCGTGGCTCGT | 59 |
| ciclev10005095m | GAGGAAGCAAGTGGAGCGAA | CCTGTGAGTTGGACGAGGGT | 60 |
| ciclev10012453m | GCTGCGTGTTCCTCTAACGA | TGCCCATATCTCCATCTCCA | 58 |
| ciclev10031065m | CAGGCTTATGTCCCGTTTGG | TTCTGAAAGCGGGAGAGTGC | 59 |
| ciclev10001658m | GGGGGATTACACGACCAAGA | GTTGCCTGCAAAGACCAACA | 60 |
| ciclev10022473m | TATGGGAATCGCAACTGTGG | CGGCAGAACATCAAAGTCCA | 58 |
| ciclev10000886m | CACAAATTGAACATGGCTTTGC | CCTCGAGGTCCTGGTGGTAA | 58 |
| ciclev10031272m | ATCATCAGCAGCAACCAGCA | GGCGTGCCTGTAGGATTTCA | 58 |
| ciclev10025448m | ATGGAGGCTTGCAAAGGAGA | AATGGGCTGATCTTGCATCC | 58 |
| ciclev10001521m | GTCACGCTGCTTCCAATACG | GCGTAATCCTCCTGCTCGTC | 58 |
| ciclev10008930m | CCCAAGCTGTCCCGTAAAGA | GCAACATGGCTAGGGCTCA | 58 |
| ciclev10000895m | TCTGCCGCTGAAGATTGCTT | ACCATAATCTGCCCACGCAT | 58 |
| ciclev10009761m | CCAGGAGGAACTAGCACCCA | CCACTTGAACCCATCATCCAG | 59 |
| ciclev10019989m | GGTTCAATGGGTTTGCCGAT | AATCACCTTCTGCCCGGCTA | 58 |
| ciclev10030153m | ATGGTGGGGTGAATGGCTCT | GAGACCCGTGACAACATGCC | 58 |
| ciclev10015042m | AAGCATTGTGCATGGTGTCC | TCGTTGACTTTGACCCTCCA | 59 |
| ciclev10025382m | GCTATTACATCCCGGCGAAA | GAACCTCGATGGCCTAAACG | 58 |
| ciclev10020010m | GGCTGGATAATCAGGCAAGC | ATTCCAAGCCTAGGGCCAAT | 58 |
| ciclev10031286m | GCGAAGAAACATGGTGCAGA | GGCTTCAGCTTTGAGCCTGT | 58 |
| ciclev10031134m | TGGTGCCATTGTCATCTTGC | ACATTTGGTGCCACACCCAT | 58 |
| ciclev10015582m | AAGGGCATGAGAATGGACACA | TCACCGGAACGCTCATTAGG | 59 |
| ciclev10015700m | CTGGATTCATGTCGCTCCAA | GGGACCGAAATCCTGTTCTG | 58 |
| ciclev10012786m | ATGTGCAGCAAGATGCAAGG | TCCTGGTGGAACACATTTGC | 58 |
| ciclev10031193m | AGGAGGATTTGTCCGGGTCT | TCCACAGCCTTCCAGTCAAA | 59 |
| ciclev10005570m | ACGGTAACGGTGGGTAGTGG | ATATCCCCTTTTCGCCATCC | 59 |
| CiOSC-V2 | GGATCCAGAGGTTCCCCAATCTTGTGA | TCTAGAGGGTCACCTCCATTCCATCT | 56 |
| CiOSC-qPCR | CTTTTGGCCTGGTGACTATGGT | CATTTCCCTTCGATGTTCCACT | 59 |
| CiGAPC2-qPCR | TCTTGCCTGCTTTGAATGGA | TGTGAGGTCAACCACTGCGACAT | 58 |
| TRV1-qPCR | TTGGGTTGCTACTGATTCGACT | CTGTAAGGACCATCATACTTCGC | 58 |
| TRV2-qPCR | TGTCAGTGATCGCAGTAGAATG | CAGTCACTTTCCCGTACGGT | 58 |
| CiGAPDH | GGAAGGTCAAGATCGGAATCAA | CGTCCCTCTGCAAGATGACTCT | 58 |
| β-actin | CCAAGCAGCATGAAGATCAA | ATCTGCTGGAAGGTGCTGAG | 58 |

File S1 Amino acid sequences of oxidosqualene cyclases (OSCs) family in plants.

File S2 Amino acid sequences of cytochrome P450s (CYP450s).

File S3 Amino acid sequences of UDP-Glucuronosyl transferases (UGTs).
