# Supplementary material for: Identification of Putative Genes Involved in Limonoids Biosynthesis in Citrus by Comparative Transcriptomic Analysis
Source: Front Plant Sci. 2017 May 12;8:782. doi: 10.3389/fpls.2017.00782 (PMC5427120; doi:10.3389/fpls.2017.00782)
Supplement: Supplementary file 2 [file Data_Sheet_2.DOC]

>CAS1(Abies magnifica)AF216755.1 GI:12004572

MWKLKIAEGGSPWLQTLNNHVGRQVWEFDPKAGTSEDHLAVEKARVDFYNKRFIQHHSADLLMRLQCGGENPLSPLPAQVKLESANDITEEVIQTTLVRAIRFYATIQAHDGHWPGDYGGPMFLMPGLVIALYVTGALNAVLSEMHKKEICRYLYNHQNEDGGWGLHIEGHSTMFGTVLNYVTLRLLGQAPDGGQGAMEKGCAWILDHGGATAIPSWGKMWLSVLGVFDWTGNNPLPPEMWLLPYFLPTHPGRMWCHCRMVYLPMSYIYGRRFVGPLTGIVMSLREELYTVPYEKIDWNQARSMCAKEDLYYPHPFLQDILWGTLHKVVEPALMHWPGSMLRERALQSVMKHIHYEDENTRYICIGPVNKVLNMLCCWVEDSNSEAFKRHLARVVDYLWVAEDGMKMQGYNGSQLWDTAFATQALISTNLLDDCGPLLKKAHIYIERSQVQEDCPGDLNFWYRHISNGAWPFSTRDHGWPISDCSSEGLKAALALSQLPQDIVGKPIPSQRIFDCVNLMLSMQNSDGGFATYELTRSYPWLEKINPAETFGDIVIDYSYVECTSAITQALVSFKKLYPEHRHKEIETCILKATRYIENIQRPDGSWYGSWGVCFTYGTWFGVLGLAAAGKTYQNCSNIRKACEFLLSKQLPSGGWGESYLSCQEKVYTHLEGGRSHIVSTAWAMLALIYAGQALRDPKPLHRAAIVLVNYQMENGDFPQQEIMGVFNRNCMISYSAYRNIFPIWALGEYCRHVLQS

>ACX(Adiantum capillus-veneris)gi|160081608|dbj|AB368375.1|

MWTLKIADGDSGSTWLHTLNEHTGRQTWHFDPDAGSPSDLLAVENARREFFENRFTKKHSADLLMRMQYAKRNPLPPLPNPVKVNDQSKLPEQNVVDTLKRAVLFYSTIQAEDGHWAGDYGGPMFLMPGLVIALYVTGSLNVVLSEAHKKEMVRYLYNHQNKDGGWGLHIEGHSTMFGSVLSYVTLRLLGQELSDGEDQAMERGRAWILQHGGATTIPSWGKFWLSVLGTFEWAGNNPLPPEIWLLPYFLPIHPGRMWCHCRMVYLPMCYIYGNRFTGKITETVLALRKELFKVPYEDIDWNKARNECAKEDLYYPHPMIQDVLWATLHKLVEPALMNWPCSSLRKKALDTVIKHVHYEDENTRYICIGPVNKVLNMLCCWIEDPNSEAFKCHLPRIPDYLWVAEDGMKMQGYNGSQLWDTSFAVQALISTGLLETCGPMLKKAHHFIDRSQVRNDCPGDLQFWYRHISKGAWPFSTRDHGWPISDCTAEGFKAALALSQLPSDIVGESLQAERFYDAVNTMLSYQNGNGGVATYELTRSYPWLELINPAETFGDIVIDYQYVECTSAVIQALAAFKKLYPKHRTEEVNACIQHAAKYIESIQREDGSWYGSWGVCFTYAGWFGVIGLLSAGRTYESETLKKACNFLLSKKLSSGGWGESYLSCQDKVYTNLPNDRPHVVHTSWAMLALLYAGQAERDPRPLHEAATVLINSQLENGDYPQQEITGVFNRNCMISYSAYRNIFPIWALGEYRRRVLSH

>CAS1(Arabidopsis thaliana)AT2G07050.1

MWKLKIAEGGSPWLRTTNNHVGRQFWEFDPNLGTPEDLAAVEEARKSFSDNRFVQKHSADLLMRLQFSRENLISPVLPQVKIEDTDDVTEEMVETTLKRGLDFYSTIQAHDGHWPGDYGGPMFLLPGLIITLSITGALNTVLSEQHKQEMRRYLYNHQNEDGGWGLHIEGPSTMFGSVLNYVTLRLLGEGPNDGDGDMEKGRDWILNHGGATNITSWGKMWLSVLGAFEWSGNNPLPPEIWLLPYFLPIHPGRMWCHCRMVYLPMSYLYGKRFVGPITSTVLSLRKELFTVPYHEVNWNEARNLCAKEDLYYPHPLVQDILWASLHKIVEPVLMRWPGANLREKAIRTAIEHIHYEDENTRYICIGPVNKVLNMLCCWVEDPNSEAFKLHLPRIHDFLWLAEDGMKMQGYNGSQLWDTGFAIQAILATNLVEEYGPVLEKAHSFVKNSQVLEDCPGDLNYWYRHISKGAWPFSTADHGWPISDCTAEGLKAALLLSKVPKAIVGEPIDAKRLYEAVNVIISLQNADGGLATYELTRSYPWLELINPAETFGDIVIDYPYVECTSAAIQALISFRKLYPGHRKKEVDECIEKAVKFIESIQAADGSWYGSWAVCFTYGTWFGVKGLVAVGKTLKNSPHVAKACEFLLSKQQPSGGWGESYLSCQDKVYSNLDGNRSHVVNTAWAMLALIGAGQAEVDRKPLHRAARYLINAQMENGDFPQQEIMGVFNRNCMITYAAYRNIFPIWALGEYRCQVLLQQGE

>CS1(Avena strigosa)gi|15866701|emb|AJ311790.1|

MWRLKIAEGGGDPWLRTKNAHVGRQVWEFDPEAGDPEALAAVEAARRDFAAGRHRLKHSSDRLMRIQFEKENPLKLDLPAIKLEENEDVTEEAVSTSLKRAISRFSTLQAHDGHWPGDYGGPMFLMPGLLITLYVTGSLNTVLSPEHQKEIRRYLYNHQNEDGGWGLHIEGPSTMFGSALTYVSLRLLGEGPESGDGAMEKGRNWILDHGGATYITSWGKFWLAVLGVFDWSGNNPLPPEIWMLPYRLPIHPGRMWCHCRMVYLPMCYVYGKRFVGKITPLILELRNELYKTPYSKIDWDSARNLCAKEDLYYPHPLIQDILWATLHKFVEPVMMHWPGNKLREKALNHVMQHVHYEDENTRYICIGPVNKVLNMLTCWIEDPNSEAFKLHIPRVHDYLWVAEDGMKMQGYNGSQLWDTAFAVQAITATGLIDEFAPTLKLAHNFIKNSQVLDDCPGDLSYWYRHISKGAWPFSTADHGWPISDCTAEGLKAALLLSKISPEIVGEPVEVNRLYDAVNCLMSWMNNNGGFATYELTRSYAWLELINPAETFGDIVIDYPYVECTSAAIQALTSFKKLYPGHRRKDVDNCINKAANFIESIQRSDGSWYGSWAVCFTYGTWFGVKALVAAGRTFKSSPAIRKACEFLMSKELPFGGWGKSYLSCQDQVYTNLEGKHAHAVNTGWAMLTLIDAGQAERDPTPLHRAAKVLINLQSEDGEFPQQEIMGVFNKNCMISYSQYRDIFPVWALGEYRCRVLAAGK

>CASBPX1(Betula platyphylla)gi|18147589|dbj|AB055509.1|

MWKLKIGAETARGDGGGGGGSETWLRSLNNHLGRQIWEFHPELGTQEELQQIDDARRRFWERRFERRHSSDLLMRIQFAKENPSSANIPQVKIKDTEEVREEAVGMTLRRAINFYSTIQADDGHWPGDYGGPMFLIPGLVITLSITGTLNAFLSKEHQCEICRYLYNHQNEDGGWGLHIEGPSTMFGTALNYITLRLLGEPEDGMGAVEKARKWILDHGGATAITSWGKMWLSVLGVYEWSGNNPLPPEVWLCPYLLPCHPGRMWCHCRMVYLPMSYLYGKRFVGPITSTIQSLRKELYTVPYHEIDWNKARNDCAKEDLYYPHPLVQDILWASLYYAYEPIFMYWPAKRLREKALDTVMQHIHYEDENTRYICIGPVNKVLNMLCCWAEDPNSEAFKLHLPRILDYLWIAEDGMKMQGYNGSQLWDTTFAVQAIISTNIAEEYGQTLRKAHEYIKDSQVLEDCPGDLNFWYRHISKGAWPFSTADHGWPISDCTAEGLKAVILLSQFPSETVGKSVDVKRLYDAVHVILSLQNTDGGFATYELTRSYHWLELINPAETFGDIVIDYPYVECTSAAIQALTLFKKLHPGHRREEIENCIAKAAEFIENIQASDGSWYGSWGVCFTYAGWFGIKGLVAAGRTYKNCSSIHKACDYLLSKELASGGWGESYLSCQDKVYTNLKDNRPHIVNTGWAMLALIDAGQAERDPTPLHRAARILINSQMENGDFPQEEIMGVFNKNCMISYSAYRNIFPIWALGEYRCRVLKAL

>CASBPX2(Betula platyphylla)gi|18147591|dbj|AB055510.1|

MWKLKIAEGGSPWLRTLNNHVGRQVWEFDPKLGSPEELAEIERARETSLKVRFEKKHSSDLLMRIQFAKENPRGAVLPQVKVNETEDVTEEMVTRMLRRAISFHSTLQAHDGHWAGDYGGPMFLMPGLVITLSITGALNTVLSEEHKKEMCRYLYNHQNKDGGWGLHIEGPSTMFGTVLSYVTLRLLGEGANDGQGAIERGRKWILDHGSATAIISWGKMWLSVLGAFEWSGNNPLPPEIWLLPYMLPVHPGRMWCHCRMVYLPMSYLYGKRFVGPITPTVMSLRKELYSVPYHEIDWNQARNLCAKELYYPHPLVQDILWASLHKLVEPVLMRWPGKRLREKALRTVLEHIHYEDENTRYICIGPVNKVLNMLCCWVEDPNSEAFKLHLPRINDYLWIAEDGMKMQGYNGSQLWDTAFAVQAIISTNLFEEYGPTLEKAHMYIKKSQVREDCPGDLDFWYRHISKGAWPFSTADHGWPISDCTAEGLKAALLLSKIPPDVVGEPLVEERLYDAVNVILSLQNADGGFATYELTRSYPWLELINPAETFGDIVIDYNYVECTSAAIQALTSFKKSYPKHREEEVDVCIKRAAMFTEKIQASDGSWYGSWGVCFTYGTWFGVKGLVAAGKNFNDCFGIRKACDFLLSKQLPSGGWGESYLSCQNKVYSHVEGNRSHVVNTGWAMLALIEAGQAERDPTPLHRAARVLINSQMENGDFPQEEIMGVFNRNCMITYAAYRNIFPIWALGEYRCRVLQAP

>CAS1(Chlamydomonas reinhardtii)gi|158283862|gb|EDP09612.1|

MWKFISAGTTGGPLLRSLNGNKGRQTWEYDPAAGTPEQRAEAERLREEFTANKDKHHHSGDELLRLQSADRIRAKKHSPPSGPVPDAPDAERVEEHLKGAISFYECLQQEDGHFPGDYGGPMFLLPGLVITLYTCGALDQIFSPAHKKEALRYLHNHQNEDGGFGLHIEGGSTMFGTGLNYVMARLLGLAAEEPLCVKAREWMHARGGATYITSWGKFWLAVLGVYSWDGMNPLTPEMWLLPHNKWTGIGMLHPGRFWCHCRMVYLPMSYVYGKRGTCQETPLTAAIRQELYPMPYGRVDWNAARNQCAKEDLYYPHPLVQDILWWALYKAENVLQGSWLRRAALAECMKHIHYEDENTRYVDIGPVNKVINMLACWLEEPGGKAFQKHLPQGGSFSWKGCFAAGSSCCGLQPTAPVLIAAQAIAEAGLLQVSGHCLRKAHEYVEQSQVIEEAAAPLSAYYRHISKGAWPFSSRDHGWPISDCSSEGLKAALTLALLPEELVGPAISPERLYDCVNVILSYQNRDGGMATYENTRSFHWLEILNPAETFGDIIVDYSYVECTSACITALCSFRKQHPGHRAAEIAASLKRAEAFIRSIQRKDGSWYGSWGVCFTYACWFGATGLAALGHTYANDEALRRCAAFLADKQRADGGWGESYLSCQDKVYSHLEGDSHVVNTAWAMLALMAVGYHKVDPEPLRRGAVFLMRMQQPSGDWPQQHISGVFNRNCMITYANYRNIFPIWALGHFRRLVLLGEEEIKINS

>CsOSC1(Costus speciosus)gi|18147770|dbj|AB058507.1|MWRLKIAEGGGPWLRTKNNHVGRQVWEFDPSLGTPEEIAEVERVREAFRETRFEKKHSADLLMRLQFAKENPLEMNYPIIKIEEHEDVTEELVVTSLRKAISRVSTLQAHDGHWPGDYGGPMFLMPGLIITLYVTGALNTVLTSEHQKEIRRYLYNHQNEDGGWGLHIEGESTMFGSALTYVILRLLGEGPDDGDGAMEKGRKWILDHGSATAITSWGKMWLSVLGVFDWSGNNPLPPEMWLLPYFLPVHPGRMWCHCRMVYLPMSYIYGKRFTGPITPLILSLRKELFNLPFDQLDWNKARNECAKEDLYYPHPFIQDVLWASLHKFVEPILMHWPGSKLREKAVNTAMQHVHYEDENTRYICIGPVNKVLNMLCCWIEDPNSEAFKLHLPRVMDYLWLAEDGMKMRGYNGSQLWDTAFTVQAIISTDLFEEFGLALTKAHEFIKKTQVLEDCPGDLNFWYRHISKGAWPFSTADHGWPISDCTSEGLKAALLLSKISPEIVGDPLDGKSLYDAVNVILSLMNNDGGFATYELTRSYAWLEIINPAETFGDIVIDYPYVECTSAAIQALTLFKKTYPGHRREEIDNCIRKSARFIEKIQLADGSWYGSWGVCFTYGIWFGMKGLLAAGRTYETSSCIRKACDFLLSKQVASGGWGESYLSCQNKVYTNLEGNRAHAVNTGWAMLALIDAGQGERDPKPLHRAAKVLINMQMENGEFPQQEIMGVFNKNCMISYSEYRNIFPIWALGEYRRRVLCSQFH

>CPX(Cucurbita pepo)gi|50896400|dbj|AB116237.1|

MWQLKIGADTVPSDPSNAGGWLSTLNNHVGRQVWHFHPELGSPEDLQQIQQARQHFSDHRFEKKHSADLLMRMQFAKENSSFVNLPQVKVKDKEDVTEEAVTRTLRRAINFYSTIQADDGHWPGDYGGPMFLIPGLVITLSITGALNAVLSTEHQREICRYLYNHQNKDGGWGLHIEGPSTMFGSVLNYVTLRLLGEEAEDGQGAVDKARKWILDHGGAAAITSWGKMWLSVLGVYEWAGNNPLPPELWLLPYLLPCHPGRMWCHCRMVYLPMCYLYGKRFVGPITPIIRSLRKELYLVPYHEVDWNKARNQCAKEDLYYPHPLVQDILWATLHHVYEPLFMHWPAKRLREKALQSVMQHIHYEDENTRYICIGPVNKVLNMLCCWAEDPHSEAFKLHIPRIYDYLWIAEDGMKMQGYNGSQLWDTAFAVQAIISTELAEEYETTLRKAHKYIKDSQVLEDCPGDLQSWYRHISKGAWPFSTADHGWPISDCTAEGLKAVLLLSKLPSEIVGKSIDEQQLYNAVNVILSLQNTDGGFATYELTRSYRWLELMNPAETFGDIVIDYPYVECSSAAIQALAAFKKLYPGHRRDEIDNCIAEAADFIESIQATDGSWYGSWGVCFTYGGWFGIRGLVAAGRRYNNCSSLRKACDFLLSKELAAGGWGESYLSCQNKVYTNIKDDRPHIVNTGWAMLSLIDAGQSERDPTPLHRAARVLINSQMEDGDFPQEEIMGVFNKNCMISYSAYRNIFPIWALGEYRSRVLKPLK

>CAS1(Dioscorea zingiberensis)gi|145651384|emb|AM697885.1|

MWRLKIAEGGNPWLRTTNNHVGRQVWEFDPNLRTPEELAEVERAREAFHQHRFEKKHSSDLLMRLQFAKENPLELTLPQVKVRDDEDVTEEAVTTTVRRAISRHSTLQAHDGHWPGDYGGPMFLMPGLVIALYVTGALNTVLSPEHQREICRYLYNHQNKDGGWGLHIEGHSTMFGSALTYITLRLLGEKTEGGDGAMQRGRKWILDHGGATFITSWGKFWLSVLGVFDWSGNNPLPPEVWMLPYFLPIHPGRMWCHCRMVYLPMSYIYGKRFVGPITPLIQSLRKELYNLSYDQINWNLARNQCAKEDLYYPHPLVQDILWASLHKVVEPILLRWPGSRLREKALHSTMQHIHYEDENTRYICIGPVNKVLNMLCCWVEDPNSEAFKFHLPRIYDYLWVAEDGMKMQGYNGSQLWDTAFTVQAIVATDLSEEFGPPLKKAHDYIKNTQVLEDCPGDLSFWYRHISKGAWPFSTADHGWTISDCTAEGLKASLLLSRISPEIVGEPVDAKRLYNAVNVILSLMNEDGGFATYELTRSYAWMEIINPAETFGDIVIDYPYVECTSAAIQALTSFKKLYPGHRREEIECCIKKAVSFIEKIQKPDGSWYGSWAVCFTYGTWFGVLGLIAGGKTYQNSPCIRKACDFLLSKELPSGGWGESYLSCQDKVYTNLEGNRPHAVNTSWVMLALIGAGQAERDPMPLHRGAKVLINMQSENGEFPQQDIMGVFNRNCMISYSAYRNIFPIWALGEYRRQVLPYLKH

>CAS1(Glycyrrhiza glabra)gi|4589851|dbj|AB025968.1|

MWKLKIAEGGSPWLRTVNNHVGRQVWEFDPKLGSPEDLLEIEKARQNFHDNRFTHKHSADLLMRIHFAKENPMNEVLPKVRVKDIEDVTEETVKTTLRRAINFHSTLQSHDGHWPGDYGGPMFLMPGLVITLSITGALNAVLTEEHRKEICRYLYNHQNKDGGWGLHIEGPSTMFGSVLNYVALRLLGEGPNDRQGEMEKGRDWILGHGGATFITSWGKMWLSVLGVYEWSGNNPLPPEIWLLPYVLPIHPGRMWCHCRMVYLPMSYLYGKRFVGPITPTILSLRKELYTIPYHDIDWNQARNLCAKEDLYYPHPLVQDILWASLHKFLEPILMHWPGKKLREMAIKTAIEHIHYEDDNTRYLCIGPVNKVLNMLCCWVEDPNSEAFKLHLPRIYDYLWIAEDGMKMQGYNGSQLWDTAFTAQAIISSNLIEEYGPTLRKAHTYIKNSQVLEDCPGDLSKWYRHISKGAWPFSTADHGWPISDCTAEGLKAVLLLSKIAPEIVGEPLDAKRLYDAVNVILSLQNEDGGFATYELTRSYTWLELINPAETFGDIVIDYPYVECTSAAIQALTSFKKLYPGHRREEIQCCIEKAASFIEKTQASDGSWYGSWGVCFTYGTWFGVKGLIAAGKSFNNCSSIRKACEFLLSKQLPSGGWGESYLSCQNKVYSNVESNRSHVVNTGWAMLALIDAEQAKRDPTPLHRAAVYLINSQMENGDFPQQEIMGVFNKNCMITYAAYRNVFPIWALGEYRHRVLQSQ

>KdCAS(Kalanchoe daigremontiana)gi|300807981|gb|HM623872.1|

MWKLKIADAGGSQWLRSVNNHIGRQIWDFDPALGSPEELAQIEDARDNFARHRFDKKHSADLLMRFQLTKENPQSDLLPKVNIGKIEDITEDAVTNTLRRAINFHSTTQAHDGHWPGDYGGPLFLMPGLVITLSITGALNAVLSKEHKKEMCRYLYNHQNEDGGWGLHIEGPSTMFGSVLNYVTLRLLGEDVNGGDGEIERARKWILDHGGATAITSWGKMWLSVLGVFEWCGNNPLPPEMWLFPYYLPVHPGRMWCHCRMVYLPMSYLYGKRFVGPITPTVLSLRKELFTVPYHEIDWNEARSLCAKEDLYYPHPVVQDILWATLHKVVEPVLLNWPGKKLREKALCSAIEHIHYEDENTRYICIGPVNKVLNMLCCWVEDPNSEAFKLHIPRLYDYLWIAEDGMKMQGYNGSQLWDTAFSVQAIVATKLVEEFSSTISKAHEFMKNSQVLEDYPGDLSYWYRHISKGAWPFSTADHGWPISDCTAEGLKVVLKLSQFPAELVGAPLSAKLVYNAVNVILSLQNIDGGFATYELTRSYSWMELLNPAETFGDIVIDYPYVECTSAALQSLVLFKKLHPEHRKEEVELCIKKAAAFIEKIQESDGSWYGSWAVCFTYGTWFGVLGLVAAGRNYKNSPSIRKACDFLLSKQLASGGWGESYLSCQNKVYTNIPGGRSHVVNTGWAMLALIGAGQAERDPVPLHRAAKFLIESQLENGDFPQQEIMGVFNKNCMISYAAYRNIFPIWALGEYRCKVLNASRGQMKT

>KcCAS(Kandelia candel)gi|152962679|dbj|AB292609.1|

MWRLKIAEGGDPWLRTINNHVGRQAWEFDPSLVGSPEDIADIENARRNFTINRFRHRHSADLLMRLQFAKENRLPEVLPKVTVKDDERVTEQAVTVALRRTLDYFSTIQAHDGHWPGDYGGPMFLMPGLVITLSVAGALNAILSREHQGEICRYLYNHQNEDGGWGLHIEGPSTMFGSVLNYVTLRLLGEGANDGEGAMERRRNWILTHGGATHITSWGKMWLSVLGVFEWRGNHPLPPEIWLLPYLLPVHPGRMWCHCRMVYLPMSYLYGKRFVGPITPTVLSLRKELFTVPYHDIDWNEARNLCAKEDLYYPHPLVQDLLWAFLDKAIEPILMHWPGKKLREKALQSVMEHVHYEDENTRYICIGPVNKVLNMLCCWVEDPNSEAFKLHLPRIYDYLWIAEDGMKMQGYNGSQLWDLSFAVQAITATNLVEEYGPTLKKAHSFVKNSQVLEDCPGDLNSWYRHISKGAWPFSTADHGWPISDCTAEGLKAALLLSKISSEIVGEPLSENQLYDAVNVLLSLQNGDGSYATYELTRSYSWLEVINPAETFGDIVIDYPYVECTSAAIQALVSFKKSYPGHRREEIERCIRKAAMFIESIQRADGSWYGSWAVCFTYATWFGIKGLVATGKNFNNCSSIRKACDFLLSRQCASGGWGESYLSCQEKVYSNLEGNRSHVVNTAWAMLALIGAGQTERDPTPLHHAARYLINSQMENGDFPQQEIMGVFNRNCTISYSAYKVIFPIWALGEYRCRVLQAS

>OSC5(Lotus japonicus)gi|83016478|dbj|AB181246.1|

MWKLKIAEGGNPWLRSTNSHVGRQVWEFDPKLGSPQDLAEIETARNNFHDNRFSHKHSSDLLMRIQFSKENPIGEVLPKVKVKDVEDVTEEAVVTTLRRAISFHSTLQSHDGHWPGDYGGPMFLMPDLVITLSITGALNAVLTDEHRKEMCRYLYNHQNKDGGWGLHIEGPSTMFGSVLNYVTLRLLGEGPNDGQGDMEKARDWILGHGGATYITSWGKMWLSVLGVFEWSGNNPLPPEIWLLPYALPFHPGRMWCHCRMVYLPMSYLYGKRFVGPITPTILSLRKELFTIPYHDIDWNQARNLCAKEDLYYPHPLVQDILWASLHKVVEPVLMQWPGKKLREKAINSVMEHIHYEDENTRYICIGPVNKVLNMLCCWVEDPNSEAFKLHLPRIYDYLWIAEDGMKMQGYNGSQLWDTAFAAQAIISTNLIEEYGPTLRKAHTFIKNSQVLEDCPGDLNKWYRHISKGAWPFSTADHGWPISDCTAEGLKAILSLSKIAPDIVGEPLDAKRLYDAVNVILSLQNEDGGLATYELTRSYSWLELINPAETFGDIVIDYPYVECTSAAIQALTSFRKLYPGHRREEIQHSIEKAAAFIEKIQSSDGSWYGSWGVCFTYGTWFGVKGLIAAGKSFSNCSSIRKACEFLLSKQLPSGGWGESYLSCQNKVYSNLEGNRPHAVNTGWAMLALIEAEQAKRDPTPLHRAALYLINSQMENGDFPQQEIMGVFNKNCMITYAAYRSIFPIWALGEYRCRVLQAR

>LcCAS1(Luffa cylindrica)gi|6045132|dbj|AB033334.1|

MWQLKIGADTVPADPSNAGGWLSSLNNHVGRQVWHFHPELGTPEDLQQIQHARQRFSDHRFEKKHSADLLMRMQFAKNNSSFVNLPQIKVKDKEDVTEEAVSRTLRRAINFYSTIQGDDGHWPGDYGGPMFLIPGLVITLSITGALNAVLSTEHQREICRYLYNHQNKDGGWGLHIEGPSTMFGSVLNYVSLRLLGEEAEDGQGAVDKARKWILDHGGASAITSWGKMWLSVLGVYEWAGNNPLPPELWLLPYLLPFHPGRMWCHCRMVYLPMCYLYGKRFVGPITPIIRSLRKELYLVPYHEVDWNKARNECAKEDLYYPHPLVQDIVWASLHHVYEPLFMRWPAKRLREKALQCVMQHIHYEDENTRYICIGPVNKVLNMLCCWVEDPHSEAFKLHIPRIFDYLWIAEDGMKMQGYNGSQLWDTAFAVQAIMSTKLAEEYGTTLRKAHKYIKDSQVLEDCPGDLQSWYRHISKGAWPFSTADHGWPISDCTAEGLKAVLLLSKLPSEIVGKSIDEEQIYDAVNVILSLQNTDGGFATYELTRSYPWLELMNPAETFGDIVIDYTYVECTSAAIQALVAFKKLYPGHRRDEIDNCVAKAADFIESIQATDGSWYGSWGVCFTYGGWFGIRGLVAAGRRYDNCSSLRKACDFLLSKELASGGWGESYLSGQNKVYTNIKDDRPHIVNTGWAMLSLIDAGQSERDPTPLHRAARILINSQMDDGDFPQEEIMGIFNKNCMISYAAYRNIFPIWALGEYRCRVLQAP

>OSC2(Oryza sativa)gi|37990834|dbj|AK121211.1|

MWRLRVAEGGGDPWLRTKNGHVGRQVWEFDPAAGDPDELAAVEAARRGFAARRHELKHSSDLLMRMQFAKANPLKLDIPAIKLEEHEAVTGEAVLSSLKRAIARYSTFQAHDGHWPGDYGGPMFLMPGLIITLYVSGALNTALSSEHQKEIRRYLYNHQNEDGGWGLHIEGHSTMFGSALTYVSLRLLGEGPDSGDGAMEKGRKWILDHGGATYITSWGKFWLSVLGVFDWSGNNPVPPEIWLLPYFLPIHPGRMWCHCRMVYLPMCYIYGKRFVGPVTPIILELRKELYEVPYNEVDWDKARNLCAKEDLYYPHPFVQDVLWATLHKFVEPAMLRWPGNKLREKALDTVMQHIHYEDENTRYICIGPVNKVLNMLACWIEDPNSEAFKLHIPRVHDYLWIAEDGMKMQGYNGSQLWDTAFTVQAIVATGLIEEFGPTLKLAHGYIKKTQVIDDCPGDLSQWYRHISKGAWPFSTADHGWPISDCTAEGLKAALLLSKISPDIVGEAVEVNRLYDSVNCLMSYMNDNGGFATYELTRSYAWLELINPAETFGDIVIDYPYVECTSAAIQALTAFKKLYPGHRKSEIDNCISKAASFIEGIQKSDGSWYGSWAVCFTYGTWFGVKGLVAAGRTFKNSPAIRKACDFLLSKELPSGGWGESYLSSQDQVYTNLEGKRPHAVNTGWAMLALIDAGQAERDPIPLHRAAKVLINLQSEDGEFPQQEIIGVFNKNCMISYSEYRNIFPIWALGEYRRRVLAADK

>OSCPNX1(Panax ginseng)gi|3688597|dbj|AB009029.1|

MWKLKIAEGGNPWLRTLNDHVGRQIWEFDPNIGSPEELAEVEKVRENFRNHRFEKKHSADLLMRIQFANENPGSVVLPQVKVNDGEDISEDKVTVTLKRAMSFYSTLQAHDGHWPGDYGGPMFLMPGLVITLSITGVLNVVLSKEHKREICRYLYNHQNRDGGWGLHIEGPSTMFGTVLNYVTLRLLGEGANDGQGAMEKGRQWILDHGSATAITSWGKMWLSVLGVFEWSGNNPLPPETWLLPYILPIHPGRMWCHRRMVYLPMSYLYGKRFVGPITPTVLSLRKEVFSVPYHEIDWNQARNLCAKEDLYYPHPLIQDILWASLDKVWEPIFMHWPAKKLREKSLRTVMEHIHYEDENTRYICIGPVNKVLNMLCCWVEDPNSEAFKLHLPRLHDFLWLAEDGMKMQGYNGSQLWDTAFAVQAIISTNLAEEYGPTLRKAHTFMKNSQVLDDCPGDLDAWYRHVSKGAWPFSTADHGWPISDCTAEGFKAVLQLSKLPSELVGEPLDAKRLYDAVNVILSLQNSDGGYATYELTRSYSWLELVNPAETFGDIVIDYPYVECTSAAIQALTAFKKLFPGHRREEIQHSIEKAALFIEKIQSSDGSWYGSWGVCFTYGTWFGIKGLVTAGRTFSSCASIRKACDFLLSKQVASGGWGESYLSCQNKVYTNLEGNRSHVVNTGWAMLALIDAGQAERDATPLHRAAKLLINSQMENGDFPQEEIMGVFDKNCMITYAAYRNIFPIWALGEYRCRVLQGPS

>PnCAS(Polypodiodes niponica)gi|262225762|dbj|AB530328.1|

MWSLKTADNGSGSPWLRSLNEHVGRQTWHFDPHSGSSTDRLAVEKARKDFTQNRFLKKHSADLLMRMQYARENLLPPLPDQVKINNHTDVTEDLVANTLKRAALFYSTIQAEDGHWAGDYGGPMFLMPGLVIVLYVTGSLNVILSEAHKEEMIRYLYNHQNKDGGWGLHIEGHSTMFGSVLSYVTLRLLGEDLNSGGDQAMQRGRTWILQHGGATAIPSWGKFWLSVLGVFERTGNNPLPPEIWLLPYFLPIHPGRMWCHCRMVYLPMCYIYGKRFTGKITELVLSLREELYGIPYTDINWSKARNECAKEDLYYPHPMLQDVLWGTLHKVVEPALMHWPGSSLRSKALATVIKHVHYEDENTRYICIGPVNKVLNMLCCWVEDPNSDAFKCHLPRIYDYLWLAEDGMKMQGYNGSQLWDVSFAVQALISTELLDTCGPMLKKAHAFIDKSQVRDDCPGDLQFWYRHISKGAWPFSTRDHGWPISDCTSEGLKAALHLSLLPPEIVGGPIPPERFYDAVNTMLSYQNANGGIATYELTRSYAWLELINPAETFGDIVIDYQYVECTSAVIQALATFQKLYPKHRTEEINECIEHAAGFIESIQKEDGSWYGSWGVCFTYAGWFGILGLIAAGRSYEHSKAIQDACSFFLSKELPSGGWGESYLSCQDKVYTNLENDRAHVVHTSWAMLALMRAGQAERDPEPLHERAAAILINAQLENGDYPQEEITGVFNRNCMISYSAYRNIFPIWALGEYKRRVLSYRV

>RsCAS(Rhizophora stylosa)gi|152962677|dbj|AB292608.1|

MWRLKIAEGGDPWLRTINNHVGRQVWEFDPSLVGSPEDIADIENARRNFTINRFHHRHSADLLMRLQFAKENRLPPVLPKVAVKDDEDVTEQAVTVTLRRALDYFSTIQAHDGHWPGDYGGPMFLMPGLVIALSVTGALNAILSREHQREICRYLYNHQNEDGGWGLHIEGPSTMFGSVLNYVTLRLIGEGANDGEGAMERGRNWILNHGGATHITSWGKMWLSVLGVFEWRGNNPLPPETWPLPYLLPVHPGRMWCHCRMVYLPMSYLYGKRFVGPITPTVLSLRKELFTVPYHDIDWNDARNLCAKEDLYYPHPLVQDLLWAFLDKAIEPFLMRWPGKKLRERAFQSVMEHVHYEDENTRYICIGPVNKVLNMLCCWVEDPNSEAFKLHLPRIHDYLWIAEDGMKMQGYNGSQLWDLSFAVQAIAATNLVEEYGPTLKKAHSFVKSSQVPEDCPGVLNSWYRHISKGAWPSSTADHGWPISDCTAEGLKAARLLSKISSVIVGEPLSANRLYDAVNILLSLQNVDGGYATYELTRSYSWLEVINPAETFGDIVIDYPYVECTSSAIQGLVSFKKSYPGHRREEIERCIRKAATFIESIQKADGSWYGSWGVCFTYATWFGIKGLVAAGKNFNNCSSIRKACDFLLSRQCASGGWGESYLSCQEKVYSNLEGNRSHIVNTAWAMLALIGAGQAERDPTPLHRSARYLINSQMDNGDFPQQEIMGVFNRNCMISYSAYKDIFPIWALGEYRCRVLQAS

>LAS1(Arabidopsis thaliana)AT3G45130.1

MWRLKLSEGDEESVNQHVGRQFWEYDNQFGTSEERHHINHLRSNFTLNRFSSKHSSDLLYRFQCWKEKGKGMERLPQVKVKEGEERLINEEVVNVTLRRSLRFYSILQSQDGFWPGDYGGPLFLLPALVIGLYVTEVLDGTLTAQHQIEIRRYLYNHQNKDGGWGLHVEGNSTMFCTVLSYVALRLMGEELDGGDGAMESARSWIHHHGGATFIPSWGKFWLSVLGAYEWSGNNPLPPELWLLPYSLPFHPGRMWCHCRMVYLPMSYLYGRRFVCRTNGTILSLRRELYTIPYHHIDWDTARNQCAKEDLYYPHPKIQDVLWSCLNKFGEPLLERWPLNNLRNHALQTVMQHIHYEDQNSHYICIGPVNKVLNMLCCWVESSNSEAFKSHLSRIKDYLWVAEDGMKMQGYNGSQLWDVTLAVQAILATNLVDDYGLMLKKAHNYIKNTQIRKDTSGDPGLWYRHPCKGGWGFSTGDNPWPVSDCTAEALKAALLLSQMPVNLVGEPMPEEHLVDAVNFILSLQNKNGGFASYELTRSYPELEVINPSETFGDIIIDYQYVECTSAAIQGLVLFTTLNSSYKRKEIVGSINKAVEFIEKTQLPDGSWYGSWGVCFTYATWFGIKGMLASGKTYESSLCIRKACGFLLSKQLCCGGWGESYLSCQNKVYTNLPGNKSHIVNTSWALLALIEAGQASRDPMPLHRGAKSLINSQMEDGDYPQQEILGVFNRNCMISYSAYRNIFPIWALGEYRKLMLSL

>OSC7(Lotus japonicus)gi|108743268|dbj|AB244671.1|

MWKLRISESKEDELIRSVNNHVGRQFWEFDPDLGTEQERAQVEQARKEFNQNRFKTKNSSDLLMRLQFERENGVNMKVKNVNIQKEEDITEEVVEDTLKRALRCYSTLQAQDGFWPGDYAGAMFMLPGLVIGLSVTGALNAALSPEHQSEMKRYVLNHQNEDGGWGLHIEGPTTMFGTVLNYVAMRLLGEDIDGGDGAMKKARKWILDRGGATSIPSWGKFWLSVLGVYEWRGINPMPPELWLLPYSLPSHPGRMWCHTRLVYLSMSYLYGRRFVGPFNALVLSLRKELYTLPYHLLDWNEARNLCAKEDLSHPRPGIQNILWGLLHHVGEPLLTHKLFSRLRQKALHHVMEHIHNEDEASNYICIGPVNKVLNMICCWLEDPNSQAFKYHISRIKDYLWVAEDGMKMQGYGGSQLWDVAFSVQAILATNLDDEYGSMLKRANEFIKCSQITTNSSSNPSAWYRHISKGSWGFSTPDNGWPVSDCTAEGLKAAILLSNFPSETVGKAMETEKLYDAVSWVLSMQNENGGFASYELTRSYAWLEKINPVETFRDIMIDYQYVECTSAAIQGLALFTQRYPEYRRREIDSCIAKAARYIESTQLADGSWYGSWGICYTYATWFGIKGLIAASKSYQESKSIRRACEFLLSKQLLSGGWGESYLSCELKVYTNLEGNKSHLVNTAWAMLALIEGGQAERDPTPLHRAAKVLINSQMENGEFPQQEIMGVYNQTGVVNYSAYRNIFPIWALGEYRNRVLLCPGKVSKNKSN

>OSCPNZ1(Panax ginseng)gi|3688601|dbj|AB009031.1|

MWKLILSQGDPGLKSVNNHIGRQFWEFDPNLGTPEERAHIDKLRQQFHNNRFRVKHSSDLLMRYQFEREKSRKLGDDDEVKSGSEGEITTSSSGVEGVKMALRRALKFYSTIQADDGHWPGDYGGPLFLLPGLVIGLYVMGVMDTILAKEHQREMCRYIYNHQNVDGGWGLHIEGCSTMLCTALNYITLRLLIRGDEEEEIRDEAANGGSLEKARRWIIDHGGATYIPSWGKFWLSILGVYEWSGNNPLPPEMWLLPYFLPLHPGRMWNHCRMVYLPMSYLYGRRFVGPINSTVLSLRRELYTHPYHQINWDLARNQCAQEDLYYPHPLIQDMLWSCLHKGVERLIMQWPLSKIRQRALTTAMQHIHYEDENTSYICLGPVNKVLNMVCCWVEDPNSMANILHLSRIKDYLWVAEDGMKMKGYNGSQLWDVGFAVQAILSTGLVDEYGSMLKKAHDFIKISQVREDSPGNLSSWNRHISKGGWPFSTPDNGWPVSDCTAEGLKAALLLSNMPFDIVGEAISPVHLYDAVNWILSLQNCTGGFASYELTRSYAWLELLNPAETFGDIVIDYQYVECTSAAIQGLKSFMRLYPGYRRKEIEACIAKATNFIESIQLPDGSWYGSWGICYTYGTWFGIKGLVAAGRTNRNCYSIRRACDFLLSKQLGSGGWGESYLSCQNKVYTSIEGNISHVANTGWAMLALIEAGQAQRDPSPLHRAAKVLMNSQMKNGVFPQQEIVGVFNKNCMISYSAYRNIFPIWALGEYLNRVLQPSRNILKTLNVV

>OSCBPW(Betula platyphylla)gi|18147593|dbj|AB055511.1|

MWKLKIAEGGPGLVSGNDFIGRQHWEFDPDAGTPQERAEVEKVREEFTKNRFQMKQSADLLMRMQLRKENPCQPIPPPVKVKETEVITEEAVITTLRRSLSFYSSIQAHDGHWPGESAGPLFFLQPFVMALYITGDLNTIFSPAHQKEIIRYLYNHQNEDGGWGFHIEGHSTMFGSALSYIALRILGEGLEDGEDGAMAKSRKWILDHGGLVAIPSWGKFWVTVLGLYEWSGCNPLPPEFWFLPDIFPIHPGKMLCYCRLVYMPMSYLYGKRFVGPITGLIQSLRQELYNEPYHQINWNKARSTVAKEDLYYPHPLIQDLLWGFLHHVAEPVLTRWPFSMLREKALKAAIGHVHYEDENSKYLCIGSVEKVLCLIACWAEDPNGEAYKLHLGRIPDNYWVAEDGLKIQSFGCQMWDAGFAIQAILSCNLNEEYWPTLRKAHEFVKASQVPENPSGDFKAMYRHINKGAWTFSMQDHGWQVSDCTAEGLKVAILFSQMPPDLVGEKIEKERLYDAVNVILSLQSSNGGFPAWEPQRAYGWLEKFNPTEFFEDTLIEREYVECTSPAVHGLALFRKFYPRHRGTEIDSSIYRGIQYIEDVQEPDGSWYGHWGICYTYGTWFAVGALAACGRNYKNCPALRKSCEFLLSKQLPNGGWGESYLSSQNKVWTNIEGNRANLVQTAWALLSLIDARQAEIDPTPIHRGVRVLINSQMEDGDFPQQEITGVFMRNCTLNYSSYRNIFPIWALGEYRRRVLFA

>GgLUS1(Glycyrrhiza glabra)gi|41687977|dbj|AB116228.1|

MWKLKIGEGGAGLISVNNFIGRQHWEFDPNAGTPQEHAEIERLRREFTKNRFSIKQSADLLMRMQLRKENHYGTNNNIPAAVKLSDAENITVEALVTTITRAISFYSSIQAHDGHWPAESAGPLFFLQPLVMALYITGSLDDVLGPEHKKEIVRYLYNHQNEDGGWGFHIEGHSTMFGSALSYVALRILGEGPQDKAMAKGRKWILDHGGLVAIPSWGKFWVTVLGAYEWSGCNPLPPELWLLPKFAPFHPGKMLCYCRLVYMPMSYLYGKKFVGPITALIRSLREELYNEPYNQINWNTARNTVAKEDLYYPHPLIQDMLWGFLYHVGERFLNCWPFSMLRRKALEIAINHVHYEDENSRYLCIGSVEKVLCLIARWVEDPNSEAYKLHLARIPDYFWLAEDGLKIQSFGCQMWDAAFAIQAILACNVSEEYGPTLRKAHHFVKASQVRENPSGDFNAMYRHISKGAWTFSMHDHGWQVSDCTAEGLKAALLLSEMPSELVGGKMETERFYDAVNVILSLQSSNGGFPAWEPQKAYRWLEKFNPTEFFEDTMIEREYVECTGSAMQGLALFRKQFPQHRSKEIDRCIAKAIRYIENMQNPDGSWYGCWGICYTYGTWFAVEGLTACGKNCHNSLSLRKACQFLLSKQLPNAGWGESYLSSQNKVYTNLEGNRANLVQSSWALLSLTHAGQAEIDPTPIHRGMKLLINSQMEDGDFPQQEITGVFMRNCTLNYSSYRNIFPIWAMGEYRRQVLCAHSY

>bAS(Artemisia annua)gi|167987428|gb|EU330197.1|

MWRLKIAEGRNDPYLYSTNNFVGRQIWEFDPNYGTPEERAEVEQARVDFWNHRHEVKPSSDVLWRMQFLREKGFEQTIPQVKIEDGEEISYEKATTTLRRSVNFFAALQADDGHWPAENAGPLYFMQPLVICLYITGHLNTVFPAEYRKEILRYIYCHQNEDGGWGFHIEGHSTMFCTTLSYICMRLLGEGRDGGLDGACTKARKWILDHGSVTTIPSWGKTWLSILGVCEWAGTNPMPPEFWILPSFLPMYPAKMWCYCRLVYMPMSYLYGKRFVGPITPLILQLRDELYAQPYDEIKWRSIRHLCAKEDLYYPHPLLQDLMWDSLYVFTEPVLNHWPFNKLREKALQTTMKHIHYEDENSRYITIGSVEKALCMLACWVEDPNGVCFKKHIARIPDYLWVAEDGMKMQSFGSQEWDAGFAIQALMATDLTDEIGSTLMKGHEFIKASQVKDNPSGDFKSMHRHISKGSWTFSDQDHGWQVSDCTAEALKCCLLFATMPPEIVGEKMKPEQLNDAVNVILSLQSKNGGLAAWEPAGSSEWLEILNPTEFFADIVIEHEYVECTSSAIQALVMFKKKYPGHRKKEIENFLLGSSGYLEKIQMEDGSWYGNWGVCFTYGTWFALGGLSAVGKTYDNCPAIRKAVKFLLETQLEDGGWGESYKSCPEKKYIPLEGGRSNLVHTAWAMMGLIHSRQAERDATPLHRAAKLLINSQLETGDFPQQEIAGVFMKNCMLHYALYRNIYPMWALADYRKQVLPQLKGT

>OXA1(Aster sedifolius)gi|60203058|gb|AY836006.1|

MWRMNIAKGGNDPYLFSTNNYVGRQIWEFDPNYGTPEELAEVEQARAEFWNNRHKVKTSSDVLWRMQFLREKGFKQTIPQVKIEDGEEITDEKAKITLRRAVNLFSALQADDGHWPAENAGPQYFMQPLVMCLYVTGHLNSVFTEEYRKEILRYMYCHQNEDGGWGFHIEGHSIMFCTTLSYICMRLLGEGPDGGLNGACAKARKWILDHGTATANPSWGKTWLSILGLCEWSGTNPMPPEFWILPSFLPMHPAKMWCYCRLVYMPMSYLYGKRFVGPITPLILELRNEIFLQPYHEINWKSIRHVVAKEDIYYPHPLLQDLMWDSLYIFTEPLLNRWPFNKLREKALRTTMNHIHYEDENSRYITIGSVEKALCMLSCWVEDPNGICFKKHLARVPDYLWVGEDGMKMQSFGSQEWDAGFAIQALLATDLTEEIGSTLKKGHEFIKASQVKDNPSGDFKSMHRHISKGSWTFSDQDHGWQVSDCTAEGLKCCLLFSNMPPEIVGEHMKPEQLKDAVNVILSLQSKNGGLSAWEPAGSSDWLEYLNPTEFFADIVIEHEYVECTSASMQALVLFKKLYPGHRRKEIENFLPNACRYLENIQMPGGSWYGNWGVCFTYGTWFALGGLTSIGKTYENCPAIRKGVKFLLETQLKDGGWGESYKSSPEKKYVPLEGGRSNLVHTAWALMGLIHSRQEERDPTPLHRAAKLIINSQLENGDFPQQEIAGVFMKNCMLHYALYRDIYPMWALADYRKHVLPKLKRI

>GgbAS1(Glycyrrhiza glabra)gi|6730968|dbj|AB037203.1|

MWRLKIAEGGKDPYIYSTNNFVGRQTWEYDPDGGTPEERAQVDAARLHFYNNRFQVKPCGDLLWRFQILRENNFKQTIASVKIGDGEEITYEKATTAVRRAAHHLSALQTSDGHWPAQIAGPLFFLPPLVFCMYITGHLDSVFPEEYRKEILRYIYYHQNEDGGWGLHIEGHSTMFCTALNYICMRILGEGPDGGQDNACARARKWIHDHGGVTHIPSWGKTWLSILGVFDWCGSNPMPPEFWILPSFLPMHPAKMWCYCRLVYMPMSYLYGKRFVGPITPLILQLREELFTEPYEKVNWKKARHQCAKEDLYYPHPLLQDLIWDSLYLFTEPLLTRWPFNKLVREKALQVTMKHIHYEDETSRYITIGCVEKVLCMLACWVEDPNGDAFKKHLARVPDYLWVSEDGMTMQSFGSQEWDAGFAVQALLATNLVEEIAPTLAKGHDFIKKSQVRDNPSGDFKSMYRHISKGSWTFSDQDHGWQVSDCTAEGLKCCLLLSMLPPEIVGEKMEPERLYDSVNVLLSLQSKKGGLSAWEPAGAQEWLELLNPTEFFADIVVEHEYVECTGSAIQALVLFKKLYPGHRKKEIENFIANAVRFLEDTQTADGSWYGNWGVCFTYGSWFALGGLAAAGKTFANCAAIRKAVKFLLTTQREDGGWGESYLSSPKKIYVPLEGSRSNVVHTAWALMGLIHAGQAERDPAPLHRAAKLIINSQLEEGDWPQQEITGVFMKNCMLHYPMYRDIYPMWALAEYRRRVPLPSTPVCLT

>OSC1(Lotus japonicus)gi|83016473|dbj|AB181244.1|

MWKLKVADGGKDPYIFSTNNFVGRQTWEYDPDAGTPEERAQVEEARQDFYNNRYKVKPCGDLLWRFQVLRENNFKQTIPSVKIEDGEEITYEKATTTLKRAAHHLAALQTSDGHWPAQIAGPLFFQPPLVFCMYITGHLNSVFPEEYRKEILRYIYVHQNEDGGWGLHIEGHSTMFCTALNYICMRMLGEGPDGGQDNACARARKWILDHGGVTHIPSWGKTWLSILGIFDWKGSNPMPPEFWILPSFLPMHPAKMWCYCRLVYMPMSYLYGKRFVGPITPLILQLREELFTQPYEKVNWKKARHQCAKEDIYYPHPLIQDLMWDSLYLFTEPLLTRWPFNKLVREKALEVTMKHIHYEDENSRYITIGCVEKVLCMLACWVEDPNGDAFKKHLARIPDYLWVSEDGMCMQSFGSQEWDAGFAVQALLATNLVDELGPTLAKGHDFIKKSQVRDNPSGDFKNMHRHISKGSWTFSDQDHGWQVSDCTAEGLKCCLLLSMLPPDIVGEKMEPECLFDSVNLLLSLQSKKGGLAAWEPAGAQEWLELLNPTEFFADIVVEHEYVECTGSAIGALVLFKKLYPGHRKKEIENFISEAVRFLEDTQTADGSWYGNWGVCFTYGSWFALGGLAAAGKTYANCAAIRKAVKFLLTTQRGDGGWGESYLSSPKKIYVPFEGNRSNVVHTAWALMGLIHSGQAERDPTPLHRAAKLLINSQLEEGDWPQQEITGVFMKNCMLHYPMYRDIYPMWALAEYRRRVPLPSTAV

>bAS1(Medicago truncatula)gi|27475607|emb|AJ430607.1|

MWKLKIGEGKNEPYLFSTNNFVGRQTWEYDPEAGSEEERAQVEEARKNFYDNRFKVKPCGDLLWRFQVLRENNFMQTIDGVKIEDGEEITYEKATTTLRRGTHHLAALQTSDGHWPAQIAGPLFFMPPLVFCVYITGHLDSVFPREHRKEILRYIYCHQNEDGGWGLHIEGHSTMFCTALNYICMRILGEGPDGGQDNACARARNWIRAHGGVTYIPSWGKTWLSILGLFDWLGSNPMPPEFWILPSFLPMHPAKMWCYCRLVYMPMSYLYGKRFVGPITPLILQLREELHTQPYEKINWTKSRHLCAKEDIYYPHPLIQDLIWDSLYIFTEPLLTRWPFNKLVRKRALEVTMKHIHYEDENSRYLTIGCVEKVLCMLACWVEDPNGDAYKKHLARVQDYLWMSEDGMTMQSFGSQEWDAGFAVQALLAANLNDEIEPALAKGHDFIKKSQVTENPSGDFKSMHRHISKGSWTFSDQDHGWQVSDCTAEGLKCCLLLSMLPPEIVGEKMEPERLYDSVNVLLSLQSKKGGLAAWEPAGAQEWLELLNPTEFFADIVVEHEYVECTGSAIQALVLFKKLYPGHRKKEIENFISEAVRFIEDIQTADGSWYGNWGVCFTYGSWFALGGLAAAGKTYTNCAAIRKAVKFLLTTQREDGGWGESYLSSPKKIYVPLEGSRSNVVHTAWALMGLIHAGQAERDPTPLHRAAKLLINSQLEEGDWPQQEITGVFMKNCMLHYPMYRDIYPLWALAEYRRRVPLPSTAV

>OSCPNY1(Panax ginseng)gi|3688599|dbj|AB009030.1|

MWKLKIAEGNKNDPYLYSTNNFVGRQTWEFDPDYVASPGELEEVEQVRRQFWDNRYQVKPSGDLLWRMQFLREKNFRQTIPQVKVGDDEAVTYEAATTTLRRAVHFFSALQASDGHWPAENSGPLFFLPPLVMCVYITGHLDTVFPAEHRKEILRYIYCHQNEDGGWGLHIEGHSTMFCTTLSYICMRILGEGPDGGVNNACARGRKWILDHGSVTAIPSWGKTWLSILGVYEWIGSNPMPPEFWILPSFLPMHPAKMWCYCRMVYMPMSYLYGKRFVGPITPLILQLREELYGQPYNEINWRKTRRVCAKEDIYYPHPLIQDLLWDSLYVLTEPLLTRWPFNKLREKALQTTMKHIHYEDENSRYITIGCVEKVLCMLVCWVEDPNGDYFRKHLARIPDYIWVAEDGMKMQSFGSQEWDTGFSIQALLDSDLTHEIGPTLMKGHDFIKKSQVKDNPSGDFKSMYRHISKGSWTFSDQDHGWQVSDCTAEGLKCCLIFSTMPEEIVGKKIKPERLYDSVNVLLSLQRKNGGLSAWEPAGAQEWLELLNPTEFFADIVIEHEYVECTSSAIQALVLFKKLYPGHRKKEIDNFITNAVRYLEDTQMPDGSWYGNWGVCFTYGSWFALGGLAAAGKTYYNCAAVRKAVEFLLKSQMDDGGWGESYLSCPKKVYVPLEGNRSNLVHTGWALMGLIHSEQAERDPTPLHRAAKLLINSQMEDGDFPQQEISGVFMKNCMLHYAAYRNIYPLWALAEYRRRVPLPSLGT

>OSCPSY(Pisum sativum)gi|8918270|dbj|AB034802.1|

MWRLKIAEGGNDPYLFSTNNFVGRQTWEYDPEAGSEEERAQVEEARRNFYNNRFEVKPCGDLLWRFQVLRENNFKQTIGGVKIEDEEEITYEKTTTTLRRGTHHLATLQTSDGHWPAQIAGPLFFMPPLVFCVYITGHLDSVFPPEHRKEILRYIYCHQNEDGGWGLHIEGHSTMFCTALNYICMRILGEGPDGGEDNACVRARNWIRQHGGVTHIPSWGKTWLSILGVFDWLGSNPMPPEFWILPSFLPMHPAKMWCYCRLVYMPMSYLYGKRFVGPITPLILQLREELHTEPYEKINWTKTRHLCAKEDIYYPHPLIQDLIWDSLYIFTEPLLTRWPFNKLVRKRALEVTMKHIHYEDENSRYLTIGCVEKVLCMLACWVEDPNGDAFKKHIARVPDYLWISEDGMTMQSFGSQEWDAGFAVQALLATNLIEEIKPALAKGHDFIKKSQVTENPSGDFKSMHRHISKGSWTFSDQDHGWQVSDCTAEGLKCCLLLSLLPPEIVGEKMEPERLFDSVNLLLSLQSKKGGLAAWEPAGAQEWLELLNPTEFFADIVVEHEYVECTGSAIQALVLFKKLYPGHRKKEIENFIFNAVRFLEDTQTEDGSWYGNWGVCFTYGSWFALGGLAAAGKTYTNCAAIRKGVKFLLTTQREDGGWGESYLSSPKKIYVPLEGNRSNVVHTAWALMGLIHAGQSERDPTPLHRAAKLLINSQLEQGDWPQQEITGVFMKNCMLHYPMYRDIYPLWALAEYRRRVPLP

>bAS(Polygala tenuifolia)gi|118582585|gb|EF107623.1|

MWRLKVGEGKNDPYLFSTNDYTGRQTWEFDPDAGTPEERAEVEAARQAFYDNRFQFKNCGDLLWRFQFLRDKNFKQTIPKVKVEDGQQITYEMATDTVRRAAHHLGGLQSSHGHWPAQIAGPLFFMPPLVFCLYITGHLNTVFPEEHRKEILRYIYYHQNEDGGWGLHIEGHSTMFCTALSYICMRMLGEGPEGGLNNACVRARKWILDHGGVTHIPSWGKTWLSVLGIFDWSGSNPMPPEFWILPSFLPMHPAKMWCYCRMVYMPMSYLYGKRFVGPITPLIKQLREELFTQPFEEINWKKARHQCASEDIYYPHPWVQDLIWDTLYICSEPLLTRWPFNKLIREKALQVTMKHIHYEDENSRYITIGCVEKVLCMLACWVEDPNGDAYKKHLARVPDYLWLSEDGMCVQSFGSQEWDAGFAVQALLAANLVDEIAPVLAKGHDFIKKSQVKDNPSGDFKSMHRHISKGSWTFSDQDHGWQVSDCTAEGLKVCLQMSLLPPEIVGEKMEPERLFDSVNVLFSLQSKKGGLAAWEPAGAQEWLELLNPTEFFADIVVEHEYVECTGSAIQALVLFKKLYPGHRKKEIDNFIINAVRFLEDTQTADGSWYGNWGVCFTYGSWFALGGLAAAGKTFSNCAAIRKAVHFLLTTQKEDGGWGESYLSSPKKIYVPLEISRSNVVQTAWAMMGLIHAGQADRDPTPLHRAAKLLINAQLENGDWPQQEVTGVFMKNCMLHYPMYRNIYPMWALAEYKRRVPLPSNAS

>BS(Vaccaria hispanica)gi|118175404|gb|DQ915167.1|

MWRLKIAEGANDPYLYSTNNFVGRQTWEFDTDYGTPEAIKEVEEARQDFYKNRFQVKPCGDLLWRFQFLREKNFKQTIPQVKLGDGEEVTYEAATATVKRAVNYLAAIQAEDGHWPAEIAGPQFFLPPLVFCLYITGHLNSVFNVHHREEILRSIYYHQNEDGGWGLHIEGHSTMFCTALNYICLRMLGVGPDEGDDNACPRARKWILDHGSVTHIPSWGKTWLSILGLFDWSGSNPMPPEFWILPSFMPMYPAKMWCYCRMVYMPMSYLYGKRFVGPITPLIKQLREELFNEPYEDIKWKKVRHFCAQEDLYYPHPLIQDLMWDSLYLFTEPLLTRWPFNGLIRKKALQVTMDHIHYEDENSRYLTIGCVEKVLCMLACWVEDPNGVCYKKHLARVPDYVWIAEDGLKMQSFGSQQWDCGFAVQALLASNLSLDEIGPALKKGHYFIKESQVKDNPSGDFKSMHRHISKGSWTFSDQDHGWQVSDCTAEGLKCCLVLSTMPPEIVGEKMDPERLYDSVNILLSLQSENGGLSAWEPAGAQAWLELLNPTEFFADIVIEHEYVECTGSAIQALVLFKKLYPGHRKKEIENFILKASKYLEDTQYPNGSWYGNWGVCFTYGTWFALGGLTAAGRTFSNCAAIRKGVEFLLKSQKEDGGWGESYISCPKKDFVPLEGPSNLTQTAWALMGLIYTRQMERDPTPLHRAAKLLINSQLESGDFPQQEITGVFMKNCMLHYPMYRSIYPMWALAEYRKHVPLRLN

>PNA(Panax ginseng)gi|115334601|dbj|AB265170.1|

MWKQKGAQGNDPYLYSTNNFVGRQYWEFQPDAGTPEEREEVEKARKDYVNNKKLHGIHPCSDMLMRRQLIKESGIDLLSIPPLRLDENEQVNYDAVTTAVKKALRLNRAIQAHDGHWPAENAGSLLYTPPLIIALYISGTIDTILTKQHKKELIRFVYNHQNEDGGWGSYIEGHSTMIGSVLSYVMLRLLGEGLAESDDGNGAVERGRKWILDHGGAAGIPSWGKTYLAVLGVYEWEGCNPLPPEFWLFPSSFPFHPAKMWIYCRCTYMPMSYLYGKRYHGPITDLVLSLRQEIYNIPYEQIKWNQQRHNCCKEDLYYPHTLVQDLVWDGLHYFSEPFLKRWPFNKLRKRGLKRVVELMRYGATETRFITTGNGEKALQIMSWWAEDPNGDEFKHHLARIPDFLWIAEDGMTVQSFGSQLWDCILATQAIIATNMVEEYGDSLKKAHFFIKESQIKENPRGDFLKMCRQFTKGAWTFSDQDHGCVVSDCTAEALKCLLLLSQMPQDIVGEKPEVERLYEAVNVLLYLQSRVSGGFAVWEPPVPKPYLEMLNPSEIFADIVVEREHIECTASVIKGLMAFKCLHPGHRQKEIEDSVAKAIRYLERNQMPDGSWYGFWGICFLYGTFFTLSGFASAGRTYDNSEAVRKGVKFFLSTQNEEGGWGESLESCPSEKFTPLKGNRTNLVQTSWAMLGLMFGGQAERDPTPLHRAAKLLINAQMDNGDFPQQEITGVYCKNSMLHYAEYRNIFPLWALGEYRKRVWLPKHQQLKI

>KdFRS(Kalanchoe daigremontiana)gi|300807977|gb|HM623870.1|

MWKLKIAEGGSDPYIYTTNNFVGRQIWEFDPQATDPQQLAKVEAARLNFYNHRHKIKPSSDLLWRLQFLEEKDFRQNIAQVKVEDGEEVSYEAATAALKRGVHFYSALQASDGHWPAENAGPMFFMSPLVMCLYITGHLNTIFTEEHRRETLRYIYYHQNEDGGWGFHIEGQSTMFGTVLNYICMRLLGEGPEGGQDNAVSRGRKWILDHGGATAIPSWGKTWLSIMGLCDWSGCNPMPPEFWLLPSYLPMHPAKMWCYCRMVYMPMSYLYGKRFTTHITPLILQLREELHTQPYDQINWKKVRHVCCKEDTYYPHPILQDLIWDTLYLTTEPLLTRWPLNKLIRERALKKTMKHIHYEDENSRYIVIGAVEKVLCMLACWVEDPNGDYFKKHLARVPDYFWVAEDGMKIQSFGSQHWDTAFFVQALLASDMTDEIRTTLAKAHDCIKKSQVKDNPSGDFRSMYRHISKGAWTFSDQDHGWQLSDCTAEGLKCCLLFSLMQPEVVGEAMPPERLYDSVNVLLYLQSKNGGMPGWEPAGESEWLELLNPTEFFENIVIEHEYVECTSSAVQALVLFKKLYPLHRRKEVERFITNGAKYLEDIQMPDGSWYGNWGVCFTYGAWFALEGLSAAGKTYNNCAAVRKGVDFLLNIQLEDGGWGESYQSCPDKKYVPLEDNRSNLVQTSWALMGLIYAGQADRDPTPLHRAAKLLINSQLEDGDFPQQEIAGVFKMNCTLHFAAYRNIFPIWALAVYRRFCNPNSEAISKPSK

>OsPS(Oryza sativa Japonica)gi|32976345|dbj|AK066327.1|

MGEAVWSSLKRAISRVCNLQAHDGHWPGDYAGLMFFLPGLIITLHVSGVLNTVLSSEHQKEMRRYIYNHQNEDGGWGLHIEGHSTMLGSSLNYVALRLLGEGPNGGDGCIENGRNWILDHGGATFTTSWGKFWLSVLGVFDWSGNNPVPPELLLLPYQLPFHPGRMSSYIRMVFIPMSYIYGKRFVGPVTPVVLELRSELYNDPYDEIDWNKARTQCAKEDMYYPRSSKLDMFWSFLHKFIEPVLLRWPGRKLREKALATSMRNVHYEDECTRYICFGGVPKALNILACWIEDPSSEAFKCHIARVYDYLWIAEDGMKMQIYDGSQVWDAGLTVEALVATDLVKELGPTLKRAHSFLKNSQLLDNCPRDFNRWYRHISKGGWTFTTADDGWQVSDCTATALKACLLLSRISPEIVGEPLEIDAQYDAVNCLMSLMNDNGGFSAFELVRSNTWLEHINPTEAFGRVMIEYPYVECTSSSIQCLALFKKLHPGHRKEEVENCISKGANFIESSQRSDGSWYGSWGICFTYATWFAVTGLVSAGRTLGNSATVRKACDFLLSKQLPSGGWGESYLSCHDEVYTNLKGNRPHGTHTAWAMIALIDAGQAERDPVPLHRAAKALLNLQLEDGEFPQQEIVGVFLQTAMISYSQYRNIFPIMALTGYRRRVLLAGNI

>SHS1(Aster tataricus)gi|340007142|dbj|AB609123.1|

MWRLKIADGGNNPYLYSTNNFIGRQTWEFDPNYGTPEERDEVEQARLHFWNHRHEIKPSGDTLWRMQFIREKKFKQTIPQVKIEDDEEISYDKVTATMRRSVHLLEALLADDGHWPAENSGPSFFIQPLVMCLYITGHLNSVFPAEHRKEILRYVYSHQNKDGGWGLHMEGHSIMFGTTLSYICMRLLGEGPDGGLNGACTRARKWILDHGGAIANPSWGKVWLSILGVHEWVGCNPLPPEFWLFPSFLPMSPGKMWSYCRLVFMPMSYLYGRRFVGPITPLVLQLRKELYAQPYNDIKWKSSRHVCAKEDIYYPHPLLQDLMWDSLYILTEPLLTRWPFNKLRKKALATTMRHIHYEDENSRYITIGSVEKILCMLACWDEDPNGVCFKKHLARIPDYIWVAEDGMKMQTFGSQVWDASIGIQALLATELTHDIAPILKKGHEFIKASQVRDNPSGDFKSMYRHISKGSWTFSDQDHGWQLSDCTTIGLTCCLLLSTMPPETVGEKMDPEQLKDAVNVILSLQSENGGLAAWEPAGSSNWLEMLNPIEFIEDIVIEHEYVECTGTGMEALVLFKKLYPKHRTKEVESFLTNAARYLDNTQMPDGSWYGEWGICFTYGTYYALGGLAAIEKTYENCQSIRKAVRFLLKTQGEDGGWGESYRSCAEKIYIPLDGNRSTVVHTAWAMLGLMHSKQEERDPIPLHRAAKLLINSQMENGDFPQQDTTGAFKKNCLLHYPMYRNIYTLWALAQYRKKVLRQPTGI

>StrBOS(Stevia rebaudiana)gi|224228176|dbj|AB455264.1|

MWRLKIADGNNNPYLYSTNNFVGRQTWEFDPNYGTQEERDEVEQARQHFWNNRHQFKATGDVLWRMQFIREKRFKQTIPQVKIEDGEEISYDKVTATLRRSVHLLAALQADDGHWPAENTGPMFFIQPLVICLYITGHLDRVFPKEHKKEILRYLYTQQNEDGGWGLHIEGQSIMFGTIMSYVCMRLLGEGPDGGLNGACTKARKWILDHGSVLASPSWGKIYLTILGVHEWEGCNPLPPEFWILPSIFPMHPAKMWCYCRLIYMPMSYLYGRRFVGPITPLVLQLREELYSQSYNDIKWKSTRHLVVKEDLHYPHPWLQDLMWDGLYIFTEPLLTRWPFSKLREKALKTTINHIHYEDENSRYITIGAVEKSLCMLACWDEDPDGVCFKKHLARIPDYIWVSEDGLKMQSFGSQLWDASLAIQALLATDLNHDIEPILRKGHDFIKASQVKDNPSGDFKSMYRHITKGSWTFSDQDHGWQTSDCTTEGLKCCLLLSKMSAEIVGEKMQPEQFYDAVNLILSLQCKNGGEAGWEPAGESNWLEFLNPSELFEDIVLEHDSVECTATGMQALVIFKKLYPKHRREEIEKFLKDACGYLEKVQMQDGSWYGEWGICFTYGTCFALGGMEAIGKTYENCEAIRRAVNFLLTTQRNDGGWGESYRSSPKKKYVPLEGNRSNLVQTACALMGLIRSKQEERDPTPLHRAAKLLINSQMENGDFPQEETGGVFKKNCLLHYPMYRNIYTLWALGEYRKKVLPQPTKV

>CPQ(Cucurbita pepo)gi|50896402|dbj|AB116238.1|

MWRLKVGAESVGEEDEKWVKSVSNHLGRQVWEFCADAAADTPHQLLQIQNARNHFHHNRFHRKQSSDLFLAIQYEKEIAKGAKGGAVKVKEGEEVGKEAVKSTLERALGFYSAVQTRDGNWASDLGGPLFLLPGLVIALHVTGVLNSVLSKHHRVEMCRYLYNHQNEDGGWGLHIEGTSTMFGSALNYVALRLLGEDADGGDGGAMTKARAWILERGGATAITSWGKLWLSVLGVYEWSGNNPLPPEFWLLPYSLPFHPGRMWCHCRMVYLPMSYLYGKRFVGPITPKVLSLRQELYTIPYHEIDWNKSRNTCAKEDLYYPHPKMQDILWGSIYHVYEPLFTRWPGKRLREKALQAAMKHIHYEDENSRYICLGPVNKVLNMLCCWVEDPYSDAFKLHLQRVHDYLWVAEDGMRMQGYNGSQLWDTAFSIQAIVATKLVDSYAPTLRKAHDFVKDSQIQEDCPGDPNVWFRHIHKGAWPLSTRDHGWLISDCTAEGLKASLMLSKLPSTMVGEPLEKNRLCDAVNVLLSLQNDNGGFASYELTRSYPWLELINPAETFGDIVIDYPYVECTAATMEALTLFKKLHPGHRTKEIDTAIGKAANFLEKMQRADGSWYGCWGVCFTYAGWFGIKGLVAAGRTYNSCLAIRKACEFLLSKELPGGGWGESYLSCQNKVYTNLEGNKPHLVNTAWVLMALIEAGQGERDPAPLHRAARLLMNSQLENGDFVQQEIMGVFNKNCMITYAAYRNIFPIWALGEYCHRVLTE

>bAS(Arabidopsis thaliana)gi|145337729|ref|NM_106544.4|

MWRLKIGEGNGDDPYLFTTNNFAGRQTWEFDPDGGSPEERHSVVEARRIFYDNRFHVKASSDLLWRMQFLREKKFEQRIAPVKVEDSEKVTFETATSALRRGIHFFSALQASDGHWPAENAGPLFFLPPLVFCLYITGHLDEVFTSEHRKEILRYIYCHQKEDGGWGLHIEGHSTMFCTTLNYICMRILGESPDGGHDNACGRAREWILSHGGVTYIPSWGKTWLSILGVFDWSGSNPMPPEFWILPSFFPVHPAKMWSYCRMVYLPMSYLYGKRFVGPITSLILQLRKELYLQPYEEINWMKVRHLCAKEDTYYPRPLVQELVWDSLYIFAEPFLARWPFNKLLREKALQLAMKHIHYEDENSRYITIGCVEKVLCMLACWVEDPNGDYFKKHLSRISDYLWMAEDGMKMQSFGSQLWDTGFAMQALLASNLSSEISDVLRRGHEFIKNSQVGENPSGDYKSMYRHISKGAWTFSDRDHGWQVSDCTAHGLKCCLLFSMLAPDIVGPKQDPERLHDSVNILLSLQSKNGGMTAWEPAGAPKWLELLNPTEMFSDIVIEHEYSECTSSAIQALSLFKQLYPDHRTTEITAFIKKAAEYLENMQTRDGSWYGNWGICFTYGTWFALAGLAAAGKTFNDCEAIRKGVQFLLAAQKDNGGWGESYLSCSKKIYIAQVGEISNVVQTAWALMGLIHSGQAERDPIPLHRAAKLIINSQLESGDFPQQQATGVFLKNCTLHYAAYRNIHPLWALAEYRARVSLP

>OSCBPY(Betula platyphylla)gi|18147595|dbj|AB055512.1|

MWRLKIADGGSDPYIYSTNNFVGRQTWEFDPQAGSPQERAEVEEARRNFYDNRYQVKPSGDLLWRMQFLKEKNFKQTIPPVKVEDGEEITYEKSTAALRRAVHFYSALQASDGHWPAENAGPLFFLPPLVMCMYITGHLNTVFPAEHQKEILRYIYYHQNEDGGWGLHIEGHSTMFCTALSYICMRILGEGPDGGQDNACARARKWILDHGGVTHMPSWGKTWLSILGIFEWIGSNPMPPEFWILPSFLPMHPAKMWCYCRMVYMPMSYLYGKRFVGPITPLILQLREELYTQPYHQVNWKKVRHLCAKEDIYYPHPLIQDLLWDSLYIFTEPLLTRWPFNKLVREKALQVTMKHIHYEDENSRYITIGCVEKVLCMLACWVEDPNGDYFKKHIARIPDYIWVAEDGIKMQSFGSQEWDTGFAIQALLASNLTDEIGPTLARGHDFIKKSQVKDNPSGDFESMHRHISKGSWTFSDQDHGWQVSDCTAEGLKCCLLFSIMPPEIVGEKMEPEQLYDSVNVLLSLQSKNGGLAAWEPAGAQEWLELLNSTEFFADIVIEHEYIECTASAMQTLVLFKKLYPGHRKKEIENFIKNAAQFLQVIQMPDGSWYGNWGVCFTYGTWFALGGLAAVGKTYNNCLAVRRAVDFLLRAQRDNGGWGESYLSCPKKEYVPLEGNKSNLVHTAWAMMGLIHAGQAERDPTPLHRAAKLIINSQLEDGDFPQQEITGVFMKNCMLHYAAYKNIYPLWALAEYRKHVPLPLGKNLNQVVNCIGQSLYKKYK

>BgbAS(Bruguiera gymnorhiza)gi|157679390|dbj|AB289585.1|

MWRIKIAEGGKDPYLYSTNNYVGRQTWEFDPDAGTPEERAEVEEARQNFYKNRYQVKPCGDLLWRLQFLGEKNFEQTIPQVRIEEGEGITYEKATRALRRTVQFFSALQASDGHWPAEIAGPLFFLPPLVMCVYITGHLDAVFPAEHRKEILRYIYYHQNEDGGWGLHIEGHSTMFCTALNYICMRIIGEGPNGGQDDACARARKWIHDHGSVTNIPSWGKTWLSILGVYDWSGSNPMPPEFWMLPSFLPMHPAKMWCYCRMVYMPMSYLYGKRFVGPITPLIQQLREELFTQPYDQINWKKTRHQCAPEDLYYPHPFVQDLIWDCLYIFTEPLLTRWPLNEIIRKKALEVTMKHIHYEDESSRYITIGCVEKVLCMLACWVEDPNGDYFKKHLARIPDYIWVAEDGMKMQSFGSQEWDTGFAIQALLATNLTDEIGDVLRRGHDFIKKSQVRDNPSGDFKSMYRHISKGSWTFSDQDHGWQVSDCTAEGLKCCLLFSMMPPEIVGEHMVPERLYDSVNVLLSLQSKNGGLSAWEPAGAQEWLELLNPTEFFADIVIEHEYVECTSSAIHALVLFKKLYPGHRKKEIDNFIVNAVRYLESIQTSDGGWYGNWGVCFTYGTWFALGGLAAAGKTYNNCLAMRKAVDFLLRIQRDNGGWGESYLSCPEKRYVPLEGNRSNLVHTAWALMALIHAGQMDRDPTPLHRAARLMINSQLEDGDFPQQEITGVFMKNCMLHYAAYRNIYPLWALAEYRRRVPLPS

>EtAS(Euphorbia tirucalli)gi|73991373|dbj|AB206469.1|

MWKLKIAEGGNDEYLYSTNNYVGRQTWVFDPQPPTPQELAQVQQARLNFYNNRYHVKPSSDLLWRFQFLREKNFKQTIPQAKINEGEDITYEKATTALRRAVHFFSALQASDGHWPAENAGPLFFLPPLVMCLYITGHLDTVFPAPHRLEILRYIYCHQNEDGGWGLHIEGHSTMFCTVLSYICMRLLGEGPNGGQDNACSRARKWIIDHGGATYIPSWGKTWLSILGVYEWSGSNPMPPEFWILPTFLPMHPAKMWCYCRMVYMPMSYLYGKRFVGPITPLILQLRQELHTQPYHHINWTKTRHLCAHEDVYYPHPLIQDLMWDSLYIFTEPLLTRWPFNKIIRKKALEVTMKHIHYEDENSRYITIGCVEKVLCMLACWAEDPNGVPFKKHLARIPDYMWVAEDGMKMQSFGSQQWDTGFAIQALLASNLTEEIGQVLKKGHDFIKKSQVKENPSGDFKSMHRHISKGSWTFSDQDHGWQVSDCTAEGLKCCLLFSMMPPEIVGEKMDAQHLYNAVNILISLQSKNGGLAAWEPAGAQQWLEMLNPTEFFADIVIEHEYVECTASAIHALIMFKKLYPGHRKKEIENFITNAVKYLEDVQTADGGWYGNWGVCFTYGTWFAVGGLAAAGKNYNNCAAMRKAVDFLLRTQKQDGGWGESYLSCPHKKYVPLEDNRSNLVHTSWALMGLISAGQMDRDPTPLHRAAKLLINSQLEDGDFPQQEITGVFMKNCMLHYAAYRNIYPLWALAEYRNRVPLPSTTL

>bAS1(Nigella sativa)gi|198443495|gb|FJ013228.1|

MWKLKIADATGPFSEYLYSTNNYIGRQTWEFDPDAGTPEERAEVEKARQDFHKNRFDIKPCGDVLLRLQMLKENKDRFDLSIPPVKLSENQIASYEDVTTTLRRAVRFVSAMQTSDGHWAADIGGPLYFMPPLVFALYITGTLDTIFSPEHKKEILRYMYVHQNEDGGWGFHIEGHSTMFGTTLNYICMRMLGEGPDGGEDNACARGRKWIRDHGGVTWIPSWGKTWLSILGLYEWSGCNPMPPEFWVLPSFLPFHPAKMWCYCRLVYMPMSYLYGKRFVGPITGLILTLREELHLQPYDEIRWFKSRNACAKEDLYYPHPLVQNLLWDTLNVFGETVLTRWPMSKLRDKALQVTMKHIHYEDENSRYITIGCVEKSLCMLACWVEDPDSDAFKKHLARVQDYLWVAEDGMRMQSFGSQNWDTGFALHGLLASNLHDEIWDTLNKGHDYVKQSQVKDNPSGDFRSMHRHLSKGSWTFSDQDHGWQVSDCTGEGLMVCLLMSQLSPEYVGPKMEPEGLYDSVNILLSLQSKNGGLAAWEPVSAPEWLEVINPTEFFQDIVIEHEYVECTASGIAALELFKKLYPGHRKKEIESFIAKAVHFLEETQMPDGSWYGNWGICFIYGTWFALRGLAAVGNNCNNSPTVRKACDFLLSTQLESGGWGESYKSCPEKKFIPLEDKRTNLVHTAWALMGLINGGQAQRDPTPLHRAVKVLINGQMENGDFPQQEITGVFMKNCMLHYAAFRNMFPLWALGEYRRKCLSS

>BgLUS(Bruguiera gymnorhiza)gi|157679392|dbj|AB289586.1|

MWRLKIAEGGNNPYIYSTNNFVGRQTWEFDPEAGTPEERAQVEEARENFWRDRFLIKPSSDLLWRFQFLSEKKFKQRIPQVKVQDGEEITREIATTALRRSVHLVSALQASDGHWCAENSGPMFFVPPMVFSLYITGHLNAVFSAEHCKEILRYIYCHPNEDGGWGLHIEGHSAMFSTVLNYNWLGKLGEGRDGGKDNACERARRRILDHGSATAISSWGKTWLAILGVYEWDGCNPMPPEFWAFPTFFPIHPARMLCYCRLTYMAMSYLYGKKFVGPITPLILQLREEIYNEPYDQINWSRMRHLCAKEDNYYAHTLTQIILWDAIYMLGEPLLKRWPFNKLREKALKITMDHIHYEDENSQYITIGSVEKPLLMLACWHEDPNGDAFKKHLARIPDYVWLGEDGIKIQSFGSQVWDTSFVLQALIASNLPSETGPTLEKGHNFIKNSQVTQNPSGDFRRMFRHISKGSWTFSDKDHGWQVSDCTAESLKCCLLFSMMPPELVGEKMGPQRMYDAVNVIISLQSKNGGCSAWEPAGAGSWMEWLNPVEFLADLVIEHEYVECTSSSLQALVLFKKLYPEHRRKEIEIFILNAVRFTEEIQQPDGSWYGNWGICFLSGTWFGLKGLAAAGKTYYNCTAVRKGVEFLLQTQRDDGGWGESYLSCPKKIYVPLEGNRSNLVQTALAMMGLILGGQGERDPTPLHRAAKLLINSQTELGDFPQQELSGCFMRNCMLHYSEYRDIFPTWALAEYCKLFPLPSKND

>KdLUS(Kalanchoe daigremontiana)gi|300807979|gb|HM623871.1|

MWKLKIADGGSNPYIFTTNNFVGRQIWEFDPQATDPQQLAKVEAARLDFYHNRYKLKPNSDLLWRMQFLEEKAFTQTIPQVKVEDGEEVSYEAVTAALRRGVHLYSALQASDGHWPAENAGPMFFMPPMVMCLYITGHLNAIFTEEHRSETLRYIYYHQNEDGGWGFHIEGHSTMFGTVLNYICMRLLGEGPEGGQDNAVSRGRKWILDHGGATSIPSWGKTWLSIMGLCDWSGCNPMPPEFWLLPSYLPMHPGKMWCYCRMVYMPMSYLYGKRFTARITPLILQLREEIHIQPYDQIDWKKVRHVCCKEDMYYPHPLLQDLLWDTLYLTTEPLLTRWPLNKLIRKRALQTTMKHIHYEDENSRYITIGCVEKVLCMLACWVEDPNGDYFKKHLARIPDYLWIAEDGMKMQSFGSQHWDTAFSIQALLASNMAEEIGITLAKGHDFIKKSQVKDNPSGDFKGMYRHISKGAWTFSDQDHGWQVSDCTAEGLKCCLLFSMMQPEVVGESMAPESLYNSVNVLLSLQSQNGGLPAWEPAGAPEWLELLNPTEFFENIVIEHEYVECTSSAVQALVLFKKLYPLHRRKEVERFITNGAKYLEDIQMPDGSWYGNWGVCFTYGAWFALEGLSAAGKTYNNCAAVRKGVDFLLNIQLEDGGWGESYQSCPDKKYVPLEDNRSNLVQTSWALMGLIYAGQADRDPTPLHRAAQLLINSQLEDGDFPQQEITGVFQRNCMLHYAAYRNIFPLWALAEYRRQIQLHSEATKMV

>RcLUS(Ricinus communis)gi|82468802|gb|DQ268869.1|

MWRIKIAEGGNNPYIYSTNNFQGRQIWVFDPNAGTPEEQAEVEEARQNFWKNRFQVKPNSDLLWQLQFLREKNFKQKIPKVKVEDGEEITSEIAAAALRRSVHLFSALQASDGHWCAENGGLLFFLPPLVFAVYITGHLNTVFSPEHRKEILRYIYCHQNEDGGWGIHIEGHSTMFCTVLNYICMRILGEARDGGIENACERGRKWILDHGGATGISSWGKTWLSILGVYEWDGTNPMPPEFWAFPSSFPLHPAKMFCYCRITYMPMSYLYGKRFVGPITPLILQIREEIYNEPYNKIKWNSVRHLCAKEDNYFPHPTIQKLLWDALYTFSEPLFSRWPFNKLREKALKITMDHIHYEDHNSRYITIGCVEKPLCMLACWIEDPHGEAFKKHLARIADYIWVGEDGIKMQSFGSQTWDTSLALQALIASDLSHEIGPTLKQGHVFTKNSQATENPSGDFRKMFRHISKGAWTFSDKDQGWQVSDCTAESLKCCLLFSMMPPEIVGEKMEPEKVYDSVNVILSLQSQNGGFTAWEPARAGSWMEWLNPVEFMEDLVVEHEYVECTSSAIQALVLFKKLYPRHRNKEIENCIINAAQFIENIQEPDGSWYGNWGICFSYGTWFALKGLAAAGRTYENCSAIRKGVDFLLKSQRDDGGWAESYLSCPKKVYVPFEGNRSNLVQTAWAMMGLIYGGQAKRDPMPLHRAAKLLINSQTDLGDFPQQELTGAFMRNCMLHYALFRNTFPIWALAEYRRHVLFPSAGFGFGFTNNL

>MRN1(Arabidopsis thaliana)gi|145358767|ref|NM_123624.3|

MWRLRIGAEARQDPHLFTTNNFAGRQIWEFDANGGSPEELAEVEEARLNFANNKSRFKASPDLFWRRQFLREKKFEQKIPRVRIEDAEKITYEDAKTALRRGVLYYAACQANDGHWPSEVSGSMFLDAPFVICLYITGHLEKIFTLEHVKELLRYMYNTQNEDGGWGLDVESHSVMFCTVLNYICLRILGVEPDHDGQKSACARARKWILDHGGATYAPMVAKAWLSVLGVYDWSGCKPLPPEIWMLPSFSPINGGTLWIYIRDLLMGMSYLYGKKFVATPTALILQLREELYPQPYSKIIWSKARNRCAKEDLLYPKSFGQDLFWEGVHMLSENIINRWPLNKFVRQRALRTTMELVHYHDETTHYITGACVAKPFHMLACWVEDPDGDYFKKHLARVPDFIWIAEDGLKFQLMGMQSWNAALSLQVMLAANMDDEIRSTLIKGYDFLKQSQISENPQGDHLKMFRDITKGGWTFQDREQGLPISDGTAESIECCIHFHRMPSEFIGEKMDVEKLYDAVNFLIYLQSDNGGMPVWEPAPGKKWLEWLSPVEHVENTVVEQEYLECTGSVIAGLVCFKKEFPDHRPKEIEKLIKKGLKYIEDLQMPDGSWYGNWGVCFTYGTLFAVRGLAAAGKTFGNSEAIRRAVQFILNTQNAEGGWGESALSCPNKKYIPSKGNVTNVVNTGQAMMVLLIGGQMERDPSPVHRAAKVLINSQLDIGDFPQQERRGIYMNMLLHYPTYRNMFSLWALALYTNALRLLVS

>THAS1(Arabidopsis thaliana)gi|145334775|ref|NP_001078733.1|

MWRLRTGPKAGEDTHLFTTNNYAGRQIWEFDANAGSPQEIAEVEDARHKFSDNTSRFKTTADLLWRMQFLREKKFEQKIPRVIIEDARKIKYEDAKTALKRGLLYFTALQADDGHWPAENSGPNFYTPPFLICLYITGHLEKIFTPEHVKELLRHIYNMQNEDGGWGLHVESHSVMFCTVINYVCLRIVGEEVGHDDQRNGCAKAHKWIMDHGGATYTPLIGKALLSVLGVYDWSGCNPIPPEFWLLPSSFPVNGGTLWIYLRDTFMGLSYLYGKKFVAPPTPLILQLREELYPEPYAKINWTQTRNRCGKEDLYYPRSFLQDLFWKSVHMFSESILDRWPLNKLIRQRALQSTMALIHYHDESTRYITGGCLPKAFHMLACWIEDPKSDYFKKHLARVREYIWIGEDGLKIQSFGSQLWDTALSLHALLDGIDDHDVDDEIKTTLVKGYDYLKKSQITENPRGDHFKMFRHKTKGGWTFSDQDQGWPVSDCTAESLECCLFFESMPSELIGKKMDVEKLYDAVDYLLYLQSDNGGIAAWQPVEGKAWLEWLSPVEFLEDTIVEYEYVECTGSAIAALTQFNKQFPGYKNVEVKRFITKAAKYIEDMQTVDGSWYGNWGVCFIYGTFFAVRGLVAAGKTYSNCEAIRKAVRFLLDTQNPEGGWGESFLSCPSKKYTPLKGNSTNVVQTAQALMVLIMGDQMERDPLPVHRAAQVLINSQLDNGDFPQQEIMGTFMRTVMLHFPTYRNTFSLWALTHYTHALRRLLP

>KdGLS(Kalanchoe daigremontiana)gi|300807975|gb|HM623869.1|

MWKLKIADGGSNPYIFTTNNFVGRQIWEFDPQATDPQQLAKVEAARLDFYHNRYKLKPNSDLLWRMQFLEEKDFRQNIPQVKVEDGEEVSYEAVTAALRRGVHLYSALQASDGHWPAENAGPMFFMPPMVMCLYITGHLNAIFTEEHRSETLRYIYYHQNEDGGWGFHIEGHSTMFGTVLNYICMRLLGEGPEGGQDNAVSRGRKWILDHGGATSIPSWGKTWLSIMGLCDWSGCNPMPPEFWLLPSYLPMHPGKMWCYCRMVYMPMSYLYGKRFTARITPLILQLREEIHIQPYDQIDWKKVRHVCCKEDMYYPHPLLQDLLWDTLYLTTEPLLTRWPLNKLIRQRALQKTMKHIHYEDENSRYITIGTVEKVLCMLACWVEDPNGDYFKKHLARVPDYFWVAEDGMKIQSFGSQHWDTVFSAQALLASDMADEIGTTLAKAHYCIKESQVKDNPSGDFRSMYRHISKGSWTFSDQDHGWQLSDCTAEGLKCCLLFSLMQPEVVGEAMPPERLFDSVNILLYLQSKNGGMPGWEPAGASEWLELLNPTEFFENIVIEHEYVECTSSAVQALVLFKKLHPGHRRKEVERFITNGAKYIEDIQMPDGAWYGNWGVCFTYGAWFALGGLAAAGKTYNNCAAVRKGVDFLLRIQLEDGGWGESYQSCPDKKYVPLEDNRSNLVHTSWALMGLLCSGQADRDPNPLHRAAKLLINSQLEDGDFPQQEITGVFKMNCMLHFAAYRSIFPVWALAEYKRFCNLSSEAISKPSK

>KdTAS(Kalanchoe daigremontiana)gi|300807973|gb|HM623868.1|

MSFVWVEESKECSEQRKGSMWKLKIAQGGKDPYLYSTNNYVGRQTWEFDPEAGTPEERAEVEAARLNFYNNRYRVKPSADLLYRMQFLKEKNFKQTIPPVKVEDGEEITYETATTALKRAVHFYSALQASDGHWPAENSGPLFFLPPLVMCLYITGHLNTVFPAEHQREILRYIYYHQNEDGGWGLHIEGHSTMFCTALSYICMRILGEGPDGGLDNAVARGRKWILDHGTVTAMPSWGKTWLSIMGLFDWSGSNPMPPEFWLLPSFLPMYPAKMWCYCRMVYMPMSYLYGKRFVGPITPLILQLREELYDQPYEQVNWKQVRHECAKEDIYYPHPKIQDLLWDTLYIAIEPLLTRWPFNKLVRERALQRTMKHIHYEDENSRYITIGCVEKVLCMLACWVEDPNGDYFKKHLARVPDYIWVAEDGMKMQSFGSQQWDTGFAIQALLASNMSDEIGETLAKGHDFVKKSQVKDNPSGDFKSMHRHISKGSWTFSDQDHGWQVSDCTAEGLKCCLLFSLMPPELVGEKMEPERLYDSVNILLSLQSKNGGLAAWEPAGAPEWLELLNPTEFFADIVIEHEYVECTASAIQALVLFKKLYPGHRKKDIETFIKGAAQYIEDRQMPDGSWYGSWGVCFTYGTWFALGGLAAAGKNYDNCAAIRKGTEFLLNTQCENGGWGESYRSCPEKRYVPLEENKSNLVHTAWALMGLIHSRQAERDITPLHRAAKLLINSQLENGDFPQQEITGVFMKNCMQHYAAYRNIYPLWGIAEYRKQIPLPLR

>CAMS1(Arabidopsis thaliana)gi|22330735|ref|NM_148667.1|

MWKLKIANGNKEEPYLFSTNNFLGRQTWEFDPDAGTVEELAAVEEARRKFYDDRFRVKASSDLIWRMQFLKEKKFEQVIPPAKVEDANNITSEIATNALRKGVNFLSALQASDGHWPAENAGPLFFLPPLVFCLYVTGHLHEIFTQDHRREVLRYIYCHQNEDGGWGLHIEGNSTMFCTTLNYICMRILGEGPNGGPGNACKRARDWILDHGGATYIPSWGKTWLSILGVFDWSGSNPMPPEFWILPSFLPIHPAKMWCYCRLVYMPMSYLYGKRFVGPISPLILQLREEIYLQPYAKINWNRARHLCAKEDAYCPHPQIQDVIWNCLYIFTEPFLACWPFNKLLREKALGVAMKHIHYEDENSRYITIGCVEKALCMLACWVEDPNGIHFKKHLLRISDYLWIAEDGMKMQSFGSQLWDSGFALQALVASNLVNEIPDVLRRGYDFLKNSQVRENPSGDFTNMYRHISKGSWTFSDRDHGWQASDCTAESFKCCLLLSMIPPDIVGPKMDPEQLYEAVTILLSLQSKNGGVTAWEPARGQEWLELLNPTEVFADIVVEHEYNECTSSAIQALILFKQLYPNHRTEEINTSIKKAVQYIESIQMLDGSWYGSWGVCFTYSTWFGLGGLAAAGKTYNNCLAMRKGVHFLLTTQKDNGGWGESYLSCPKKRYIPSEGERSNLVQTSWAMMGLLHAGQAERDPSPLHRAAKLLINSQLENGDFPQQEITGAFMKNCLLHYAAYRNIFPVWALAEYRRRVPLPYEKPSTERRS

>LUP1(Arabidopsis thaliana)gi|30699379|ref|NM_179572.1|

MWKLKIGKGNGEDPHLFSSNNFVGRQTWKFDHKAGSPEERAAVEEARRGFLDNRFRVKGCSDLLWRMQFLREKKFEQGIPQLKATNIEEITYETTTNALRRGVRYFTALQASDGHWPGEITGPLFFLPPLIFCLYITGHLEEVFDAEHRKEMLRHIYCHQNEDGGWGLHIESKSVMFCTVLNYICLRMLGENPEQDACKRARQWILDRGGVIFIPSWGKFWLSILGVYDWSGTNPTPPELLMLPSFLPIHPGKILCYSRMVSIPMSYLYGKRFVGPITPLILLLREELYLEPYEEINWKKSRRLYAKEDMYYAHPLVQDLLSDTLQNFVEPLLTRWPLNKLVREKALQLTMKHIHYEDENSHYITIGCVEKVLCMLACWVENPNGDYFKKHLARIPDYMWVAEDGMKMQSFGCQLWDTGFAIQALLASNLPDETDDALKRGHNYIKASQVRENPSGDFRSMYRHISKGAWTFSDRDHGWQVSDCTAEALKCCLLLSMMSADIVGQKIDDEQLYDSVNLLLSLQSGNGGVNAWEPSRAYKWLELLNPTEFMANTMVEREFVECTSSVIQALDLFRKLYPDHRKKEINRSIEKAVQFIQDNQTPDGSWYGNWGVCFIYATWFALGGLAAAGETYNDCLAMRNGVHFLLTTQRDDGGWGESYLSCSEQRYIPSEGERSNLVQTSWAMMALIHTGQAERDLIPLHRAAKLIINSQLENGDFPQQEIVGAFMNTCMLHYATYRNTFPLWALAEYRKVVFIVN

>LUP2(Arabidopsis thaliana)gi|145337730|ref|NM_106545.3|MWKLKIGEGNGEDPYLFSSNNFVGRQTWEFDPKAGTPEERAAVEDARRNYLDNRPRVKGCSDLLWRMQFLKEAKFEQVIPPVKIDDGEGITYKNATDALRRAVSFYSALQSSDGHWPAEITGTLFFLPPLVFCFYITGHLEKIFDAEHRKEMLRHIYCHQNEDGGWGLHIEGKSVMFCTVLNYICLRMLGEGPNGGRNNACKRARQWILDHGGVTYIPSWGKIWLSILGIYDWSGTNPMPPEIWLLPSFFPIHLGKTLCYTRMVYMPMSYLYGKRFVGPLTPLIMLLRKELHLQPYEEINWNKARRLCAKEDMIYPHPLVQDLLWDTLHNFVEPILTNWPLKKLVREKALRVAMEHIHYEDENSHYITIGCVEKVLCMLACWIENPNGDHFKKHLARIPDFMWVAEDGLKMQSFGSQLWDTVFAIQALLACDLSDETDDVLRKGHSFIKKSQVRENPSGDFKSMYRHISKGAWTLSDRDHGWQVSDCTAEALKCCMLLSMMPAEVVGQKIDPEQLYDSVNLLLSLQGEKGGLTAWEPVRAQEWLELLNPTDFFTCVMAEREYVECTSAVIQALVLFKQLYPDHRTKEIIKSIEKGVQFIESKQTPDGSWHGNWGICFIYATWFALSGLAAAGKTYKSCLAVRKGVDFLLAIQEEDGGWGESHLSCPEQRYIPLEGNRSNLVQTAWAMMGLIHAGQAERDPTPLHRAAKLIITSQLENGDFPQQEILGVFMNTCMLHYATYRNIFPLWALAEYRKAAFATHQDL

>PEN1(Arabidopsis thaliana)gi|30683212|ref|NM_117622.2|

MWRLRIGAKAGNDTHLFTTNNYVGRQIWEFDANAGSPQELAEVEEARRNFSNNRSHYKASADLLWRMQFLREKGFEQKIPRVRVEDAAKIRYEDAKTALKRGLHYFTALQADDGHWPADNSGPNFFIAPLVICLYITGHLEKIFTVEHRIELIRYMYNHQNEDGGWGLHVESPSIMFCTVINYICLRIVGVEAGHDDDQGSTCTKARKWILDHGGATYTPLIGKACLSVLGVYDWSGCKPMPPEFWFLPSSFPINGGTLWIYLRDIFMGLSYLYGKKFVATPTPLILQLQEELYPEPYTKINWRLTRNRCAKEDLCYPSSFLQDLFWKGVHIFSESILNRWPFNKLIRQAALRTTMKLLHYQDEANRYITGGSVPKAFHMLACWVEDPEGEYFKKHLARVSDFIWIGEDGLKIQSFGSQLWDTVMSLHFLLDGVEDDVDDEIRSTLVKGYDYLKKSQVTENPPSDHIKMFRHISKGGWTFSDKDQGWPVSDCTAESLKCCLLFERMPSEFVGQKMDVEKLFDAVDFLLYLQSDNGGITAWEPADGKTWLEWFSPVEFVQDTVIEHEYVECTGSAIVALTQFSKQFPEFRKKEVERFITNGVKYIEDLQMKDGSWCGNWGVCFIYGTLFAVRGLVAAGKTFHNCEPIRRAVRFLLDTQNQEGGWGESYLSCLRKKYTPLAGNKTNIVSTGQALMVLIMGGQMERDPLPVHRAAKVVINLQLDNGDFPQQEVMGVFNMNVLLHYPTYRNIYSLWALTLYTQALRRLQP

>CSOSC2(Costus speciosus)gi|18147772|dbj|AB058508.1|

MWRLKVAEGSGPWLRSTNNHVGREVWEFDPSGGTPEEIAEVERARETFRDHWVEHTNSADLIMRLQFEKENPAEMKYSVIRIQEAENISKEAVDICLRKAVTRISTLQAHDGHWPGDCGAGPMFLLPGLVISLHITGALNTILTPEHQKEMRRYLYNHQNVDGGWGLYSNGPSNMIGSVLHYVTLRLLGEGANDGEGAMEKGRKWILDHGSATATSSWGKLWLSVLGVYEWAGNNPMPPEFWLLPYCVPLHPGKMWCLSRTVYLPMSYLYGKRFVGAITPIVVSLRKELYNVPYDHIDWNKARTDCAKEDQYYPHPLIQDIIWGSLHNFVEPILMRWPGSKLREKALSTVMQHIHYEDENTRYICVGPVNKALNMLCCWIDDPNSEAFKLHLPRVYDYLWLAEDGMKMKAYDGFQLWEAAFAVQAIVSTNLSEEFGPTLKKAHEFVKNSQILEDCPGDLNYWYRHISKGAWTFSTADEGWPVSDCTGEGLEAVLLLSMISPKIVGDPLDERRLYDAVNLLLSLMNKNGGFATYELTRSYAWLEIMNPADVFINIVVDHQYVECTSSAIQPLALFKKLYPGHRQEEIDNCIMKAARFIERTQRADGSWYGSWGVCFTYATWFGVKGLVAAGRTYENSCYIQKACNFLLSKQEASGGWGESFLSCRKKVYINLEGNKTHAVNTSWAMLALIAAGQGERDPKPLHRAAKALINMQMENGDFPQQEMMGNFMGGSSLNYPLYRNIFPTWALGEYRNHIFHSMT

>KcMS(Kandelia candel)gi|116292145|dbj|AB257507.1|

MWRLKIAEGGDNPYIYSTNNFLGRQTWEFEPEAGTPEERAQVEEARQNFWRDRFRIKPCSDLLWRFQFLREKKFKQIIPQGKVQDGEEITRDIATTALRRSVHLLSALQASDGHWCAENSGPMFYVPPMVFALYITGHLTTVFSAEHCKEILRYIYCHQNEDGGWGLHIEGHSTMFCTVLNYICMRILGEGRDGGKDNACERARKWILDHGSATAISSWGKTWLAILGVYEWDGCNPMPPEFWVFPTFFPIHPAKMLCYCRLTYIAMSYLYGKKFVGPITPLILQLREEIYNEPYDEINWSRMRHLCAKEDNHYPHTLTQIILWDAIYLLSEPLLKRWPWSKLRKKALKITIDHIHYEDENSRYITIGCVEKPLNMLACWHEDPNGDAFKKHLARISDYVWLAEDGMKIQSFGSQAWDTSFVLQALIASNLLSETAPTLEKGHNFIKDSQVTENPSGDFRRMFRHISKGSWTFSDKDHGWQVSDCTAESLKCCLLFSMMPPELVGRKMEPQRVYDAVNVIISLQSKNGGCSAWEQAGAGSWMEWLNPVEFLEDLVIEHEYIECTSSSVQALVLFKKLYPEHRRKEIENFIVNAVRFIEEIQKPDGSWYGNWGICFLFGTWFGLKGLATAGKTYYNCTAVRKGVEFLLRTQREDSGWGESYLSCPKKVYVPLEGNQSNLIHTALAMMGLILSGQAERDPTPLHRALKLLINSQTELGDFPQQEISGCFMRNCMLHYSAYRDIFPMWALAEYCKLFPLPSKND

>AMY2(Lotus japonicus)gi|28194507|gb|AF478455.1|MWKLKVADGGKNPYIFSINNFVGRQTWEYDPDAGTPEERAQVEEARQDFYNNRYKVKTCGDRLWRFQVMRENNFKQTIPSVKIEDGEKVTYDKVTTTVRRAAHHLAGLQTSDGHWPAQIAGPLLFTPPLIFCMYITGHLDSVFPEVYRKEILRYTYVHQNEDGGWGLHIEGHSTMFCTVLNYICMRILGEGPDGGQDNACARARKWIHDHGGATHIASWGKTWLSILGIFDWSGTNPMPPEFWILPSFLPMHPAKMWCYCRLVYMPMSYLYGKRFVGPITPLILQLREELFTQPYEKVNWKKARHQCAKEDLYYPHPLIQDLMWDSLYLFTEPFLTRWPFNKLIRERALQVTMKHIHYEDHNSRYITIGCVEKVLCMLACWVEDPNGIAFKRHLARVPDYLWLAEDGMCMQSFGSQEWDAGFAVQALLSTNLIDELGPALAKGHDFIKNSQVKDNPSGDFKSMHRHISKGAWTFSDQDHGWQVSDCTAEGFKCCLLLSMLPPEIVGEKIEPERLFDTVNLLLSLQSKKGGFAVWEPAGAQEWLELLNPIEFFEDIVIEHELVECTGSAIGALVLFKNHYPEHRKKEIEDCIANAVRYFEDIQTADGSWYGNAGICFIYGTWFALGGLEAAGKTYANCAAIRKGVKFLLTTQSKDGGWGESYLSCPKKIYVPLEGNRSNVVQTAWALMGLIHAGQAERDPTPLHRAAKLLINSQLEDGDWPQQDITGVYVKNCTLHYPMYRNNFTTMALAEYRRRVPLPSIAV

>OSC8(Oryza sativa)gi|32980558|dbj|AK070534.1|

MWRLKIAAESGGGSGSSPLLHTGNGFLGRAVWEFDPDAGTPEERAEVARLRRDFTRHRFQRKESQDLLMRMQYAKLGHLQPDLSAVIVEDNQNVTEETILSSLRRALNQYSTLQAHDGHWPGDYSGILFIMPLLIFSMHVTGTLDVVLSLEHKREICRYIYNHQNEDGGWGTQVLGQSTMFGSCLNYATLKLLGEALHNNDALAQGRMWILSHGSATAAPQWAKIWLSVIGVYDWSGNKAIIPELWMVPHFLPIHPARFWCFVRMIYMSMAYLYGKKFVGPITPTILEIREELYNIPYSEIDWKKARDCCAKEDLRYPCSWIQDIVWTYLNKYVDPMFNVWPFNKLREISLRNLMKHIYYEDENTKYIGLCPINKALNMICCWIEDPNSDAFKRHLPRIYDFLWLAEDGMKAQVYDGCQTWETAFIVQAICSTGLVDEFSTTLEKAYGFLKNSQVLHDLPNGKSFYRHRSKGSWTLSTADNGWSVPDCTGETLQALLGLSKISPKLVGDPIKEKSLYDAVDCLLSFSNKDGTFSSYECTRTASWTEILNPSESFRNIVVDYPHVECTSSAIQGLISFTELYPGYRGVEIESCIKNAVMFIENKQQNDGSWYGTWGICFTYGAFFAIRGLIAAGRNYENSQAIRNGCKFLLSKQLSAGGWGEHYSSSEIEVYVDSGSPHAVNTSLAMLALLYSGQIERDPTPLYRAAKQLISMQLETGEFPQQEHVGCFNSSLYFNYPNYRNLYPIWALGEFWHRLVASKD

>OSCPSM(Pisum sativum)gi|44890923|dbj|AB034803.2|

MWKLKIGDGGKDRNIFSTNNFVGRQTWEFDPDAGTSQEKAQVEAARQHFYDNRFEVKACSDLLWRFQILKEKNFKQTIESVKIKDEEEISEENVAITLRRAVHHLSTLQSNDGHWPALNAGPLFYFPPLVFCMYVTGHLDSIFPYEYRKEILRYIYCHQNEDGGWGLHVEGHSIMFCTVLNYICMRILGEGPNGGKEDACARARKWIHDHGSVTHVSSWGKIWLSVLGIFDWCASNPMPPEFWMLPSFLLKHPAKMLCYCRLVYMPMSYLYGKRFVGPITPLILMLREELLTQPYEKVNWKKTRHLCAKEDLYYPHPLIQDLIWDSLYIFVEPLLTHWPFNKLLREKALQTVMKHIHYEDENSRYITIGCVEKVLCILACWVEDPNGDAFKKHLARLPDYLWVSEDGMTLHSFGSQTWDASLIIQALLATNLIEDVGPILTKAHEFIKKSQVRDNPSGDFKSMYRHISKGSWTFSDKDHGWQVSDCTAESLKCCLLLSMLPPEIVGEKMEPEMLYDSVNILLSLQGKKGGLPAWEPSEAVEWLELFNPIEFLEEIVVEREYVECTSSAIQALVLFKKLYPEHRKKEVENFIANAVRFLEYKQTSDGSWYGNWGICFTYGSWFALNGLVAAGKTYDNCAAIRKGVEFLLTTQREDGGWGESHLSSSKKIYVPLERSQSNIVQTSWAIMGLIHAGQMERDPTPLHRAVKLIINFQQEEGDWPQQELTGVFMKNCMLQYAMYRDIFPTWALAEYRRRILLASPAVAI

>Bi(Cucumis sativus)gi|698029649|gb|KM655855.1|

MWRLKVGKESVGEKEEKWIKSISNHLGRQVWEFCAENDDDDDDEAVIHVVANSSKHLLQQQRRQSSFENARKQFRNNRFHRKQSSDLFLTIQYEKEIARNGAKNGGNTKVKEGEDVKKEAVNNTLERALSFYSAIQTSDGNWASDLGGPMFLLPGLVIALYVTGVLNSVLSKHHRQEMCRYIYNHQNEDGGWGLHIEGSSTMFGSALNYVALRLLGEDANGGECGAMTKARSWILERGGATAITSWGKLWLSVLGVYEWSGNNPLPPEFWLLPYSLPFHPGRMWCHCRMVYLPMSYLYGKRFVGPITHMVLSLRKELYTIPYHEIDWNRSRNTCAQEDLYYPHPKMQDILWGSIYHVYEPLFNGWPGRRLREKAMKIAMEHIHYEDENSRYIYLGPVNKVLNMLCCWVEDPYSDAFKFHLQRIPDYLWLAEDGMRMQGYNGSQLWDTAFSIQAILSTKLIDTFGSTLRKAHHFVKHSQIQEDCPGDPNVWFRHIHKGAWPFSTRDHGWLISDCTAEGLKASLMLSKLPSKIVGEPLEKNRLCDAVNVLLSLQNENGGFASYELTRSYPWLELINPAETFGDIVIDYSYVECTSATMEALALFKKLHPGHRTKEIDAALAKAANFLENMQRTDGSWYGCWGVCFTYAGWFGIKGLVAAGRTYNNCVAIRKACHFLLSKELPGGGWGESYLSCQNKVYTNLEGNRPHLVNTAWVLMALIEAGQGERDPAPLHRAARLLINSQLENGDFPQQEIMGVFNKNCMITYAAYRNIFPIWALGEYSHRVLTE

>ATLUP2(pacid:20804871)

MWRLKIGDHRTKNDPYIFSTNNHVGRQIWEFDPDADSPEELAEVEGARLNYFNNRFNVKNSSNLIWQIQREEIQTNNSVV

KIADHGEEITLETATGALRRAVHIFSALQSSHGHWPADNSGPLFYNTPFVIYLYITGYLNSVLSSEHRKEMLRYTYNHQG

HSTMFGTVFNYICMRLLGEGPDGGENNACARARKWILDRGGAMGVTSWGKTWLSILGVYDWSGCNPMPPEFWKIPSFLPI

SPGKLNCYCRVTYMPMSYLYGKRFVGPITPLILQLREEIYTQPYTDIDWSKMRHFCAKEDIFFPHTTVQNLLWDTLYNVV

EPILNRWPLNKLREKSLEIAMNHIHYEDEASRYMTIECVEKPLNMLCCWIEDPNSDYFKKHLPRIGEYLWVGEDGMKVQS

FGSQTWDCAFAVQALLACTLTDEIGPILMKAHDFLKICQVTNNPPGDFKSMFRHMSKGAWTFSNKDHGWPVSDYTAEALL

CCLHFSLMQPEIVGEKMEPERFYDAINCILPMQSETGRVRAWEPTRAPARLELLNPIEFLDQVIIEHDKVECTASTLKAM

TLFKKLYPKHRAKEVKNLIRNAAKFIEDTQKPDGSWYGSWGICFLYGIWFAISGLAAAKKTYSNCLAIRKATEFLLKIQC

EDGGWGESYHSCPNKKYVPLDGNRSNLVQTAWAMLSLIHAGQMERNPTPLHRAAKLLINSQLQDGDFPQQ

>BAS(pacid:20799330)

MWRLKIAVGDKNSPYMFTTNNFVGRQIWEFDPNAGSPEELAEVEEARQSFYKNRHNVKPAGDLLWRLQFLREKNFKPRIP

QVKVKDGEAITYETATTAMKRAAHYFSAIQASDGHWPAENAGPMYFLPPFVFCLYITGHLNTVFTVEHRREILRYLYNHQ

HEDGGWGVHVEAPSSMFGTVFSYLCMRLLGLGPNDGENNACARARKWIRDHGGVTYIPSWGKNWLSILGIFEWSGTNPMP

PEFWILPSFVPLHPSKMWCYCRLVYMPVSYLYGKRFVGPITPLIQQLREELHTQPYNEINWRKVRHLCSKEDLYYPHPFV

QELLWDTLYLASEPLLTRWPLNKLIRQKALKETMKFIHYEDHNSRYITIGCVEKPLCMLACWVEDPNGIAFKKHLNRIAD

YIWLGEDGMKVQTFGSQTWDTALGLQALMACNIADEVESVLGKGHDYLKKAQIRDNPVGDYKGNFRHFSKGAWTFSDQDH

GWQVSDCTAEGLKCVLQLSLMPPEIVGEKMEPERLYDAVNFLLSLQDEKTGGLAVWERAGASLLLEWLNPVEFLEDLIVE

HTYVECTASAIEAFMLFKKLYPHHRKKEIENFIVKAVHYIEDEQTADGSWYGNWGICFIYGTCFALGGLQVAGKTYNNCL

AIRRAVDFLLNAQSDDGGWGESYKSCPNKIYTPLEGKRSTVVHTALAVLSLISAGQADRDPTPIHRGVKLLINSQLENGD

FPQQEIMGVFMRNCMLHYAEYRNIFPLRALAEYRKRVPLPN*

>CAMS1(pacid:20813354)

MWKLKVGEGNGEKDAYIYSTNNYAGRQIWQFDPEAGSDEERAQVEAARLHFYNNRDHLKPSADLLWRMQFLKEKKFKQTI

PQVKIKVDGDEEEITYETAATTALRRGVRFFSALQAIDGHWPAENAGPLFFLPPLVMCLYITGHLNTVFPAESEHRKEIL

RYIHYHQNEDGGWGLHIEGHSTMFCTALNYICLRILGQPPHHIACATARNWILDRGGVTLIPSWGKIWLSILGVFDWSGC

NPMPPEFWILPSFLPMHPGKMWCYCRMVYMPMSYLYGKRFVGAITPLVVELRQELYPEVEPYHKVNWGKARHLCAKEDAY

YPHPWIQDLIWDTLYVFTEPLLTRWPFSKFIREKALQVTMDHIHYEDHNSRYITIGCVEKVLCMLACWVEHPNGDSFKKH

LARIPDYLWVAEDGMKMQSFGSQQWDTGFAIQALLASNLIDEIGPVLKRGHEFIKASQVKDNPSGDFKRMYRHISKGSWT

FSDQDHGWQVSDCTAEGLKCCLLLSMMPTEIVGEKMEPERLYDSVNLLLSLQSKIGGLPAWEPVGAHKWLELLNPTEFFA

DIVIEHEYVECTSSAIQALTMFSKLYPGHRKKEIDKFIGKAIGYLEDAQMADGSWYGCWGVCFTYGTWFALGGLAAAGKT

YTNSPTVRKAVHFLLNSQTQNGGWGESFRSCPQKKYIPLEGNRSNLVQTAWAMMGLLHAGQAERDPTPLHRAAKLLINSQ

LEDGDFPQEEITGVFMRNCMLHYAAYRNIYPLWALAEYCRKLKDRKILHGFRAPWDSTSNEVNFE*

>CiOSC(Citrus)

MWKLKLSEGNCKNDPWLTSLNNHIGRQFWEFDPNLGTPEERQQVNKLRNDFTENRFLTKHSSDLLMRLQFAKENACEMKQ

LPQVKVKDDEEISDEQVVSTTLRRALRFYSSLQAEDGFWPGDYGGPLFLLPGLVMGLFVTGALNAILTVEHRREMIRYLY

NHQNRDGGWGLHIEGCSTMFCTALSYVTLRLLGETMDGGDDGAMEKARKWILDRGGVTSIPSWGKMWLSVLGVYEWSGNN

PLPPEIWLLPYFLPFHPGRMWCHCRMVYLPMSYLYGTRFVGPFNSLILSLRKELYTLPYHYIDWDHARNLCAKEDLYYPH

PMIQDILWGCLHKIGEPLVMKWPFSKLRHNALKTVMQHIHYEDENTQYICIGPVNKVLNMICCWVEDPNSEAYKRHLSRI

KDYLWIAEDGMKMQGYNGSQLWDVTFAVQAILATDLVDEYGSTLVKAFSFLKNTQVREDCTGDLNYWYRHISKGGWPFST

PDNGWPVSDCTAEGLKAAILLSQLPSDIVGVAIQENQLYDAVNVILSLQNSTGGFASYELTRSYPWLEMINPAETFGDII

IDYQYVECTSAAIQGLKSFMKSYPGHRRKEIEASIEKAIEFIESKQQADGSWYGSWGVCFTYGTWFGIKGLVACGMTYEN

SNSIRKACDFLLSKQLDSGGWGESYLSCQNKVINLILSLLRISKFVIFPGNFGKEIVNCIVGEKTIILIEIMGVFNKNCM

ISYSAYRNIFPVWALGEYLNRVLLPSKNC*
