# Supplementary material for: Identification of Putative Genes Involved in Limonoids Biosynthesis in Citrus by Comparative Transcriptomic Analysis
Source: Front Plant Sci. 2017 May 12;8:782. doi: 10.3389/fpls.2017.00782 (PMC5427120; doi:10.3389/fpls.2017.00782)
Supplement: Supplementary file 3 [file Data_Sheet_3.DOC]

>Ciclev10031065m (CYP714A1)

MLIHLPILINHQEVPSPSLSPLSIQSINKLIRSISYTTHKARESKTQKRNTKARVVMEAALVLNSAVALAIAFLLGHIYK

TVWLKCETMRRKLRMQGIKGPPPSILYGNLPEMQKIQANAAKPSSTSLASDIVAHDYTSTLFPYFEQWRKEYGPIYTYST

GMRQHLYVNQPELVKEMNQSISLDLGKPSYVTKRLAPMLGNGLSRSNGHCWAQQRKIVAPEFFMDKVKGMVGIMVESTQP

LLRKWQDCVEAEDGVNADIRVDEDLREVSADVISRASFGSSNLRGKEIFAKLRSLQKAISNQSFLFGATNFAGRFLAMKK

HDNIGNLEKEIESLIWDTVKEREQQCAGASSSSSSDLMQLILEGAVKDQSLGKDSSKRFIVDNCKNIYFAGHESTAVAAS

WCLMLLALHPEWQNHIRTELTQVCGDSLLDADSLPHLKTVTMVIQESLRLYPPAAFVSREALEDTQIGNIKIPKGLCLWT

LIPTLHRDTDIWGHDANEFKPERFAEGISKACKIPQAYVPFGVGPRLCLGRNFAMVQLKIVLSLIVSKFSFSISPNYKHS

PAFRMIVEPRYGVQILLRKI*

>Ciclev10030087m (CYP89A6)

MKEEESLGVNKEEYVLSYVDTLLDLQLHEEKKKLSEEEIVSLCSEFLSASTESTSTALQWIMANLVKHPHVQEKVYTEIR

GVVGENEEVKKEELQEMPYLKAVILEGLKRHPPGHFVLPHAVTEDFVLDDKYVIPKDGSVNFMVADMGWDPKVWEDPMAF

KLERFLNDHDQDFDITGGREIKMMPFGAGRRICPGFGLAMLHLEYFVANLVWNFEWKAVDGDEVDWTEKQEFTRLLQAAA

AANHAQITLQLFGKKSKIKHHLN*

>Ciclev10022473m (CYP71B37)

MQNPRAMKKVQLEIRSLIGGNKGFVNEDDVQELHYLMAVVKETMRLQPTVPLLIPRETIQKCVIDGYEIPAKTLVFVNAW

AIGRDPEAWENPEEFYPERFVDSCIDFKGQHFELIPFGAGRRICPGLNMGIATVDLAFANLLYKFDWEMPPGMKSQDLDF

DVLPGITMHKKNALALLAKYHE*

>Ciclev10000886m (CYP83B1)

MALLIVFLVSLPVIFYFLHLQRKLINKPKTTSALLPPGPRGLPLIGNLHQLESTNFHYQLWNLSKKYGPLMSLRLGLVQT

VVVSSVKIANEALKTYDVEFSGRPALVGQQKLTYNGLDIVFAPYNDKWKEMRKICVTHLFNASRVRHFRPVREDEVACMI

EEISSTSTSSSSPSPATVVINLSERLMSLTNSVIFRVAVGKKFENKAGERSKFHSLLDETRVVLGAFYFKDFFPFFGGFF

DKLSGIISRLENNFKEFDAFYQQLIEEHADPNRPKDQVRGDIVDVLLQVQKDRGEDQVHGFTWDNIKAVLMNVFVGATDT

SAALMTWAMTNVVKNPRVMEKAQKEVRDLIGDKGFVDEDDLEKLPYIKAILKETFRLYPPVPIIPRETTKSCVIDGYQVP

AKTLVYLNGWAISRDPEVWERPDDFDPDRFIIGDKSNIELTGQNNYELIPFGGGRRFCPGIHMGIANLELAIANLLYKFD

WEMPAEMKIQDLDFDIAPGIVMHKKHPLYLAATKYI*

>Ciclev10030448m (CYP90D1)

MDILSCMLWVLFTWVLVITLNSFLRGCKAGSRKLPPGPTPYPVIGNLLELGAKPHRSLAKLSKIYGPVMSLKFGQVTTVV

ISSATTAREILQNQDTSFCNRTVPDALRAHQHDEFSMGWLPLSTPRTNLRKICNSHIFTTQKLDANQHLRRKRIQQLLAY

AQENCRAGKAIDIGQAAFNTTLNFLWNTIFSVDLADHSSDTAREFRDMISGITVEAGKPNLSDNFPALKKLDPQGIRRRM

TKHFGKMLEVFDCLIDQRMKLRQEHGSTEYKDILDTLLNIMDDKSVEIDRNYINHLFIDLFAAGADTTSSTLEWAMTELL

CNPEALSKTRMELEQTVGKGNPIDESDIIRLPYLQAVVKETFRLHPAAPLLLPRKASTNTQVAGYTIPKDALVLVNVWAI

GRDEGIWESPCSFMPERFLGSEIDVKGRNFELIPFGAGRRICPGLPLAIRMLYLMLGSLINSFDWKFEDGITQHNMDMEE

KFGLTLNKAQPLHAIPIAI*

>Ciclev10031272m (CYP89A5)

MEFGFIILISIAVAALLKAFIGVIISSKYKTNLPPGPFNVPLIGNLRWLLKSFTEIEPILRNLHSKLGPVFTLYVGPRPA

IFIADRSLAHKALVQNGAIFADRPPPLPTWKIISSNQHDITSASYGTTWRVFRRNLSAEILHPSRVKSYRHARKWVLEIL

LNRLKSESKNGDRPVPVRLLDHFQYAMFCLLVLMCFGDKLDESQIKKIENVQRRLLLAVGKFNILNFWPRLTKIVFFKKW

IQFLQVCRDQENVLVPLIRARKKMKEEESLGVNKEEYVLSYVDTLLDLQLHEEKRKLSEEEIVSLCSEFLSAGTGSTSTV

LQWIMANLVKYQHVQEKVYTEIRGVGGENEEVKEEELQEMPYLKAVILEGLRRHPPGHFLFPHAVTEDFVLDDKYVIPKD

GSVNFMVADMGWDPKVWEDPMAFKPERFLNDHDQDFDITGSREIKMMPFGAGRRICPGFGLAMLHLEYFVANLVRNFEWK

TVDGDEVDLTEKQEFTVVMKNPLQALLSPRI*
