# Supplementary material for: Identification of Putative Genes Involved in Limonoids Biosynthesis in Citrus by Comparative Transcriptomic Analysis
Source: Front Plant Sci. 2017 May 12;8:782. doi: 10.3389/fpls.2017.00782 (PMC5427120; doi:10.3389/fpls.2017.00782)
Supplement: Supplementary file 4 [file Data_Sheet_4.DOC]

>Ciclev10020010m

MAQVKHHVACMPSPGMGHLIPHVELAKQLVLRHDISVTFLVPTIGPPSKAITSVLQGLPEHINHVLLPPVNFEEDVKAEI

QIVLAIKRSLSSVRDVFKSLVASTHLMALVVDPFGTDVFDVAREFYVPSYLYFLTNALSLSLLHYMPKLDEVISCEVRDM

EQPLKLPGFTIPIHGRDFPDPLQDRKNDAYRFMIQIRKRYSLADGILINTFMELEPGVIKALQEEPSMRSIYPIGPIIRT

VSDGELVDGSESHQCMCIRWLDNQASGSVLFVSFGSGGTLSYDQLEELALGLELSEQQFLWVVKSPDDKSASGSFFDVHS

KTDPFGFLPTGFLDRTKEQGLVVPSWAPQVEVLGHPSTGGFLTHCGWNSTLESIVHGVPLIAWPLYAEQRLNAVILSEDL

NVALRPPEYENGLIRREEIAKVIKGLMHGEDGVIIRDRMNRLKDAAAAAVSDGGSSTKTLSQLVHKWKNQN*

>Ciclev10015042m

MELETTQHIKQSISVALVPTPGMGHFIPLVELAKRLVLQHNFHVTFIIPHDGSPMQPRKQVLESLPPTSISTILLPPVSF

DDLPDHEDVSIEVRITLTLTRSLSALRETLNNLTTDQSTRLVALVVDLFGLEAFTVTREFGVPIYVFFSTTAMDLSLVFY

LPELDHKFTCDFKDLPEPVQLPGCVPICGPDLEDCLQERNSETYQSIIHLAKQYPLAAGILVNSFMDLEPGAFKALMESR

ESSFRLPPVYPVGPLILTGSINESDKTDCLKWLDDQPNGSVLFVCFGSGGTLSQKQLNELALGLEMSGQRFLWVVKCPDE

KATNATYFGVHGMKEENPFDYLPKGFLDRTKGVGLVVPSWAPQIQVLSHGSTGGFLSHCGWNSVLESIVHGVPIIAWPLY

AEQKMNAVLLTDDLKVAWRVKVNEDGLVGREEVATYARGLIQGEDGKLLRDKMRVLKDAAANALSPDGFSTKSLANVAQK

WKNLENDTN*

>Ciclev10001658m

MPLRQCLFILWLIALVLGLATTANATTTISQRFKEAPQFYNSPDCPSISSIEFELDEGEEHIFCSDEAVHVAMTLDSAYI

RGSMAAILSVLQHSSCPQNIAFHFVTSAKANASLLRATISTSFPYLKFRVHPFDDSSVSGLISTSIRSALDCPLNYARSY

LANLLPLCVRRVVYLDSDLVLVDDIAKLAATDLGNSSVLAAPEYCNANFTSYFTPTFWSNHALSLTFANRKACYFNTGVM

VIDLDRWRAGDYTTKIEEWMELQKRMRIYELGSLPPFLLVFAGNIAPVDHRWNQHGLGGDNFRGLCRDLHPGPVSLLHWS

GKGKPWARLDANRPCPLDALWAPYDLLHTPFALD*
